# Quantification of Ectopic *Fusobacterium* Colonisation in Colorectal Cancer Using a Newly Developed *nusG*-Directed PCR Method

**DOI:** 10.3390/ijms27114865

**Published:** 2026-05-28

**Authors:** Janne Becker, Anna Mertens, Meikel Duncan Rieger, Georg Conrads, Sama Rezasoltani

**Affiliations:** Division of Oral Microbiology and Immunology, Department of Operative Dentistry, Periodontology and Preventive Dentistry, Rheinisch-Westfälische Technische Hochschule (RWTH) University Hospital, 52074 Aachen, Germany; janne.becker1@rwth-aachen.de (J.B.); anna.mertens1@rwth-aachen.de (A.M.); meikel.rieger@rwth-aachen.de (M.D.R.); srezasoltani@ukaachen.de (S.R.)

**Keywords:** *Fusobacterium nucleatum* complex, colorectal cancer, N-utilisation substance G, *nusG*, endpoint PCR, quantitative PCR

## Abstract

The *Fusobacterium nucleatum* complex, which comprises oral lineage 1 (L1) strains, is strongly associated with colorectal cancer (CRC). NusG (N-utilisation substance G) is a transcription elongation factor that is universally conserved. This study aimed to develop and validate a novel *nusG*-directed polymerase chain reaction (PCR) assay to specifically and sensitively detect ectopic *Fusobacterium* L1 colonisation in clinical CRC patient samples. Four L1-specific primer pairs targeting the *nusG* gene were designed using MEGA11 software (Molecular Evolutionary Genetics Analysis, version 11.0.13) and successfully employed in 40 stool samples from CRC patients and healthy controls (HC). Additionally, five species-specific primer pairs were designed for the L1 species *F. animalis* clades 1 and 2, *F. nucleatum*, *F. polymorphum*, and *F. vincentii*, and were successfully applied to stool and saliva samples. Their specificity was verified via Sanger sequencing. Two L1-specific primer pairs (NusG5-F/NusG6-R and NusG2a-F/NusG5-R) demonstrated robust performance in our cohort, showing statistical significance (*padj* < 0.05) and a large effect size (|r| ≥ 0.5) in the difference in Ct values and absolute cell counts between CRC patients and the HC group. These primer pairs also exhibited promising preliminary diagnostic potential, with respective area under the curve (*AUC*) values of 0.909 and 0.883. However, *Fusobacterium* L1 abundance in saliva samples did not differ significantly between groups, indicating that definitive conclusions cannot be drawn due to the limited power of the salivary sub-cohort. The data indicates that *nusG*-based PCR primers could be used as reliable, non-invasive biomarkers as a complementary tool for early CRC diagnostics. While potentially applicable in the context of other *Fusobacterium*-implicated diseases, further validation in larger, ethnically diverse cohorts remains essential.

## 1. Introduction

Colorectal cancer (CRC) is the third most prevalent cancer worldwide and carries the second-highest mortality rate [1]. According to estimates by the Global Cancer Observatory (GCO), the annual incidence of new cases is projected to reach nearly 3.3 million by 2045 [2]. These figures underscore the global burden of this disease and the imperative for developing new advanced diagnostic methods and effective therapeutic approaches. Given that an estimated 12% of all cancer cases worldwide are attributable to infectious agents [3], it is crucial to investigate the role of microorganisms in carcinogenesis, especially within the oro-gastrointestinal tract.

*Fusobacterium nucleatum sensu lato* (*F. nucleatum*) is a Gram-negative, anaerobic, non-motile and non-spore-forming spindle-shaped bacterium and a frequent inhabitant of the oral cavity [4]. Its pathogenic role in periodontal disease has been well-established [5]. It also demonstrates its harmful potential outside of the mouth by its linkage to various systemic diseases, such as organ abscesses or preterm birth and stillbirth [6,7,8]. Notably, in healthy individuals, it is rarely found in the gastrointestinal tract above a cut-off of about 0.05% [9], compatible with transition through ingestion of saliva rather than true ectopic colonisation. Human oral *Fusobacterium* species are closely related to *F. nucleatum sensu stricto* (Fn) and form part of the designated lineage 1 (L1/FnL1), based on high taxonomic resolution *rpoB*-data (FrpoB-seq) [10]. This lineage, also known as the *Fusobacterium nucleatum* complex (or group), includes—besides Fn—human species such as *F. animalis* (Fa), *F. hwasookii* (Fh), *F. polymorphum* (Fp), *F. (pseudo-)periodonticum* (Fpp), and *F. vincentii* (Fv) [10]. It is noteworthy that *F. animalis* comprises two distinct clades: clade 1, which is characterised by its genetic identity to and possible renaming as *F. watanabei* and clade 2, which is expected to retain its original designation [11]. While all FnL1 species might play a role in the progression and development of CRC, recent evidence indicates that *F. animalis* clade 2 (FaC2) predominates in the microbial aetiology [12]. It was recently summarised that in 9560 samples from 11 studies, FaC2 was elevated in gut metagenomes from patients with CRC and Crohn’s disease, suggesting ecological commonalities between the two diseases [9].

FnL1-species ectopically colonise the gut with an abundance increasing from the rectum to the cecum, suggesting a gradient of influence along the colorectal segments [13]. These species are broadly associated with microbial dysbiosis, inflammation, tumour progression, and poor prognosis [14]. The association between *Fusobacterium* and CRC was first observed in 2012, when Kostic et al. and Castellarin et al. independently reported a significantly higher abundance of *F. nucleatum* in colorectal tumours compared to adjacent healthy tissue [15,16]. This discovery prompted extensive research, offering a new perspective: in the complex narrative of CRC, *F. nucleatum* may, in fact, play a protagonist role rather than simply being a supportive character. Indeed, its presence is linked to shorter patient survival [17] and chemoresistance [18]. Furthermore, *F. nucleatum* creates a proinflammatory microenvironment promoting cancer progression through recruitment of tumour-infiltrating immune cells [19]. Its outer membrane adhesins are key to its pathogenic role in CRC, enabling invasion of host cells and enrichment in tumours. Specifically, fusobacterial adhesin FadA mediates the attachment to and invasion of CRC cells by interacting with E-cadherin on the host cell surface, leading to the activation of oncogenic signalling pathways such as Wnt/β-catenin [20]. Similarly, fusobacterial lectin Fap2 facilitates the enrichment in CRC by binding to host polysaccharide Gal-GalNAc, which is frequently overexpressed in CRC cells [21]. For review of the complex *F. nucleatum* virulence traits, the reader is directed to a very recent review [11]. *F. nucleatum* (or FnL1 for broader coverage) remains a potential target for CRC detection and prevention strategies. However, specific studies on the exact number and species distribution of FnL1 are limited, and further research is needed to fully understand its pattern and role in CRC.

In this context, the *nusG* gene (encoding for the N-utilisation substance G) serves as a reliable molecular marker for detecting and differentiating bacterial species and their specific clades. The NusG protein is a universal transcription elongation factor that is found in all three domains of life and is essential for the regulation of bacterial gene expression [22]. Structurally, the protein consists of two domains: the NusG N-terminal domain (NGN) and the C-terminal domain (KOW) [23,24]. The two domains are connected by a flexible linker, enabling the protein to adopt different conformations to perform its various regulatory interactions [25]. The NGN is the most highly conserved domain of the protein and is primarily responsible for the interaction with the RNA polymerase (RNAP) [25]. The KOW shows more variety in sequence and structure and is capable of binding diverse regulatory interaction partners, such as termination factor ρ and is responsible for ensuring immediate protein synthesis by physically connecting the mRNA to ribosomes via binding to ribosomal protein S10 (NusE) [23,26]. The conserved structure of the protein dictates the various functions: by binding to the RNAP, the NGN of NusG stabilises the transcription elongation complex and promotes efficient RNA synthesis [27]. Also, NusG secures continuous forward movement of the RNAP in many bacteria, therefore serving as an anti-pausing factor [27,28]. However, it can also act as a pro-pausing factor in some species, such as *Bacillus subtilis*, through specific interaction with DNA sequences, thereby interfering with the RNAP [29]. This mechanism requires the allosteric inhibition of the so-called “swivel module” of the RNAP. Rotational changes in this region are needed during nucleotide addition. By preventing the proper movement, NusG is able to restrain the RNAP from continuing RNA synthesis [30,31]. The protein is essential for coupling transcription and translation in bacteria. In summary, the NusG protein is involved in the regulation of transcription termination and antitermination, both intrinsic and ρ-dependent, displaying its fundamental purpose in bacterial (or other domains) gene expression regulation [32,33].

Due to its highly conserved nature and broad presence, including bacteria, the *nusG* gene is an ideal target for molecular characterisation to differentiate between *F. nucleatum* species and to establish diagnostic strategies. As a single-copy gene, *nusG* allows a more accurate quantification of bacterial cell counts via qPCR, preventing issues caused by the variable copy numbers of the 16S rRNA gene. Furthermore, *nusG* provides high phylogenetic resolution, allowing for precise (sub-)species differentiation comparable to the *rpoB* gene; however, by avoiding multiple triple-wobble bases, it leads to an improved primer performance.

This study aimed to develop and evaluate a novel molecular assay targeting the *nusG* gene to achieve a highly sensitive and specific quantification/identification of ectopic L1 *Fusobacterium* colonisation within clinical CRC samples.

## 2. Results

Individual results of cell counts can be found in the Supplementary Materials, Table S1: total bacterial load in stool/saliva; Table S2: absolute fusobacterial L1 cell count and relative abundance in stool; Table S3: absolute *Fusobacterium*-species cell count and relative abundance in saliva; Table S4: absolute *Fusobacterium*-species cell count and relative abundance in stool; Table S5: *Fusobacterium* species identification in stool; and Table S6: *Fusobacterium* species identification in saliva.

### 2.1. The Phylogenetic Resolution of nusG

The primary goal was to develop a *Fusobacterium nusG* amplification and sequencing method to differentiate the *Fusobacterium* L1 content of clinical samples at the species level. The *nusG* gene is present as a single-copy gene in each genome, thus avoiding operon-to-operon variations, as is known to occur within the 16S rRNA gene. Based on all available fusobacterial and *Ilyobacter polytropus* (a close relative and Fusobacteriaceae member, used as an outgroup) *nusG* gene data from NCBI, a phylogenetic tree was constructed. The results demonstrated that *nusG* was an accurate, high-resolution taxonomic marker for *Fusobacterium* in general, and for L1 species in particular, comparable to *rpoB* (see Supplementary Materials, Figure S1). In the *nusG* tree, all the studied species formed distinct clades, outperforming the 16S rRNA gene tree and showing comparable results to the *rpoB* tree. For instance, the *nusG* gene was able to distinguish between *F. animalis* clades 1 and 2, as well as between *F. canifelinum* and *F. polymorphum*. Notably, all of the *Fusobacterium* species examined could be robustly classified into specific lineages. Consequently, the *nusG* gene has been identified as a promising target for the development of primers and a method for the amplification and differentiation of *Fusobacterium* species.

### 2.2. Primer Design for Fusobacterium L1 Detection

From a total of 11 primers initially designed and covering different NusG domains, the eight most promising primers—four forward and four reverse—were selected after extensive in silico analyses for in vitro testing. Their lengths ranged from 24 to 31 bases (Table 1). All primer sequences are given under Materials & Methods.

Next, eight primer combinations were selected to generate amplicons ranging from 87 bp (minimum) to 356 bp (maximum) in length. Other criteria were similar annealing temperature (Ta), avoidance of primer self-annealing and dimer formation, as well as coverage of NusG domains (Table 2).

While the primer combinations NusG1-F/NusG1-R and NusG1a-F/NusG1-R amplified a region within the NusG KOW domain—previously explored by Castellarin et al. [15]—all other pairings targeted the NGN or covered both regions, as the longest amplicon (356 bp) was created by NusG5-F/NusG1-R (Supplementary Materials, Figure S2).

Despite the fact that combinations such as NusG2a-F/NusG7-R were subsequently excluded from the analysis of patient samples in this context, we have included all initially chosen primer pairs here, as they may be useful in a modified study setting in the future.

### 2.3. Endpoint PCR

The eight pairs were initially assessed by endpoint polymerase chain reaction (PCR) to determine sensitivity and specificity (Table 3). An endpoint PCR was considered successful when it yielded sharp, high-intensity amplicons in positive control strains (those of L1), while showing no detectable amplification in negative control strains (non-L1).

To conclude, testing was deemed successful for seven out of eight primer pairs in terms of sensitivity and specificity, and they were incorporated into subsequent quantitative real-time polymerase chain reaction (qPCR) experiments.

### 2.4. Quantitative Real-Time PCR Applied on Reference-Strain DNA and Stool-DNA Samples

The seven remaining primer pairs were subsequently evaluated via qPCR and analysed with regard to their sensitivity (Table 4).

In summary, three pairs were excluded at this stage from further analyses due to their low sensitivity, while the remaining four combinations were selected for clinical sample analysis, starting with stool DNA.

Next, the four selected primer pairs were tested on stool samples from 25 CRC patients, and 15 HC and their sensitivity was confirmed (Table 5). Individual results can be found in the Supplementary Materials, Table S2.

### 2.5. Sanger Sequencing of Fusobacterium L1 Amplicons from Stool Samples

The purified qPCR products of 20 samples (13 different patients, including 11 CRC and 2 HC) were successfully sequenced. These samples originated from the qPCR assays of NusG1-F/NusG1-R (7 samples) and NusG2a-F/NusG6-R (13 samples) (see Clustered Heatmap under Discussion and Supplementary Materials, Table S5). The NusG5-F/NusG6-R amplicon was too short (87 bp) and lacked sufficient information for species identification, so it was not subjected to Sanger sequencing. Five distinct *Fusobacterium* species were found in the 20 samples. Seven amplicons were identified as *F. vincentii*, with all sequences identical to the NCBI reference strain SB015. Six amplicons were classified as *F. animalis* clade 2, with little sequence variance (different best-matching reference strains in NCBI). Four of the samples contained *F. animalis* clade 1 amplicons, all of which matched the same reference sequence (strain KCOM 3457) best. Two amplicons were identified as *F. polymorphum*, representing two different sequence types, and one amplicon was identifiable as *F. pseudoperiodonticum*.

### 2.6. Statistical Analysis of Results Applying L1-Specific Primers

**Figure 1:**
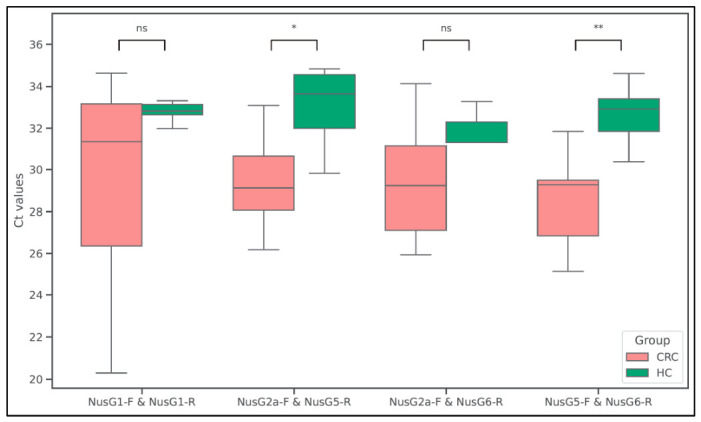
Boxplot representing the distribution of Ct values across four *Fusobacterium* L1 primer pairs in stool samples from colorectal cancer (CRC) patients versus healthy controls (HCs). Lower Ct values indicate higher bacterial abundance. The *n*-values in the plot reflect samples with detectable amplification in qPCR from a total cohort of *n*_CRC_ = 25 and *n*_HC_ = 15. Central horizontal lines represent the median, boxes indicate the interquartile range (IQR), while whiskers extend to 1.5× IQR. Outliers were omitted for visual clarity. Statistical significance between groups was assessed using the Mann–Whitney U test (ns = not significant, * = *p* < 0.05, ** = *p* < 0.01). Red = CRC, green = HC. Created with Seaborn (version 0.13.2)/Matplotlib (version 3.10.5) in Python (version 3.13.7).

Descriptive statistics, including mean, standard deviation (*SD*), median and interquartile range (*IQR*), were calculated for all qPCR data. Ct values and absolute cell count for all four primer pairs showed lower mean and median values in the CRC group compared to the HC group, consistent with a higher abundance of L1 Fusobacteria in the CRC samples (Figure 1).

To compare Ct values between CRC and HC groups, the Mann–Whitney U test was utilised. After false discovery rate (*FDR*) correction, out of the four primer pairs, two depicted statistical significance (*p_adj_* < 0.05) and a large effect size (*r*^2^ ≥ 0.25, |*r*| ≥ 0.5). Specifically, the Ct values of the primer combination NusG5-F/NusG6-R were significantly lower in the CRC group (median 29.27, *IQR* 2.64) compared to the HC group (median 32.92, *IQR* 1.56) and the effect size was the largest out of all primers (*p_adj_* = 0.003, |*r*| = 0.691). Likewise, the primer pair NusG2a-F/NusG5-R demonstrated a significant difference in CRC (median 29.12, *IQR* 2.58) versus HC (median 33.65, *IQR* 2.55) Ct values, supported by a large effect size (*p_adj_* = 0.027, |*r*| = 0.588). Regarding the other two primer pairs, NusG1-F/NusG1-R and NusG2a-F/NusG6-R, the difference between the CRC and HC groups failed to reach significance after *FDR* correction.

The Mann–Whitney U test was also applied to compare absolute fusobacterial cell counts (CFU or gene copy equivalents/µL DNA extract of stool) between CRC and HC samples. The results are consistent with previous findings: NusG5-F/NusG6-R and NusG2a-F/NusG5-R both reached statistical significance (*p_adj_* = 0.002 and *p_adj_* = 0.014, respectively), whereas NusG1-F/NusG1-R and NusG2a-F/NusG6-R failed to show a significant difference in CRC compared to HC samples regarding the absolute cell count.

**Figure 2:**
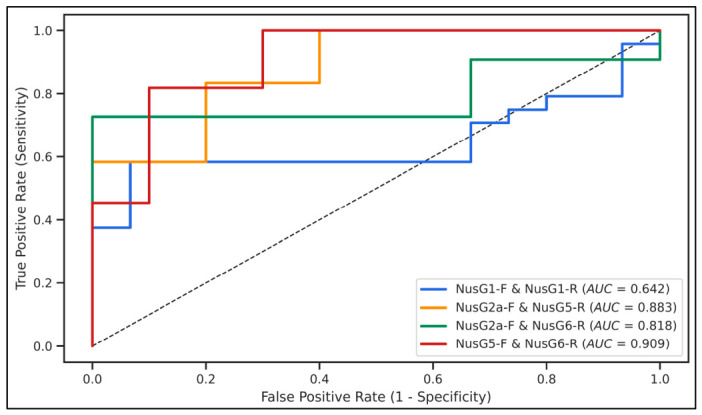
Receiver operating characteristic (*ROC*) analysis of four *Fusobacterium* L1 *nusG*-directed qPCR assays in stool samples. The curve illustrates the diagnostic performance of four different primer pairs in discriminating between colorectal cancer (CRC) patients and the healthy control (HC) group. The area under the curve (*AUC*) values for each primer pair are provided in the legend, representing the overall predictive accuracy, and the dashed diagonal line represents the performance of a random classifier (*AUC* = 0.500). Specificity and sensitivity levels were calculated across all potential Ct threshold values. Created with Seaborn (version 0.13.2)/Matplotlib (version 3.10.5) in Python (version 3.13.7).

The diagnostic performance of L1-directed primers was evaluated via receiver operating characteristic (*ROC*) analysis (Figure 2). The results were consistent with the inferential findings: The combination NusG5-F/NusG6-R demonstrated the best diagnostic performance with an area under the curve (*AUC*) value of 0.909. With a promising Ct cut-off of 29.56, it achieved a sensitivity of 0.82 and a specificity of 0.90. Similarly, the pairing NusG2a-F/NusG5-R exhibited valuable diagnostic capabilities (*AUC* of 0.883), reaching a sensitivity of 0.83 and a specificity of 0.80 at the optimal Ct cut-off of 30.98.

In contrast, the results acquired from the other two primer pairs, NusG1-F/NusG1-R and NusG2a-F/NusG6-R, indicate lower, but still potentially acceptable diagnostic performance: For NusG2a-F/NusG6-R (*AUC* of 0.818), at the optimal (by Youden’s Index) Ct cut-off of 30.81, a sensitivity of 0.73 and a specificity of 1.0 were observed. The combination NusG1-F/NusG1-R achieved the lowest accuracy (*AUC* of 0.642), demonstrating a sensitivity of 0.58 and a specificity of 0.93 at the optimal Ct cut-off of 31.59.

### 2.7. Primer Design for Detection of the Fusobacterium Species Within the L1 Lineage

The forward primer NusG2a-F was found to be optimal for the detection of *Fusobacterium* species within the L1 lineage (*F. nucleatum* complex). Although NusG5-F showed slightly higher diagnostic potential in previous experiments, NusG2a-F had the advantage of having only two degenerate bases (rather than four in the NusG5-F primer), thus minimising the risk of non-specific amplification (see Table 1 and Materials & Methods). NusG2a-F was used in combination with five additional reverse primers, each 21 bases in length (see Table 6 for PCR conditions and Materials & Methods for sequences). These reverse primers had GC contents ranging from 33.3% to 47.6% and annealing temperatures ranging from 42.4 °C to 54.7 °C; only NusG-Fnp required the inclusion of a single wobble base. The amplicons were all located in the NGN of the *nusG* gene.

Each forward–reverse pair was designed to specifically detect a different *Fusobacterium* (sub-)species, ensuring consistent amplification with a single forward primer while accommodating sequence variability for high resolution across target species.

### 2.8. Quantitative Real-Time PCR for Species-Specific Detection in Saliva and Stool Samples

The five newly designed reverse primers were tested with the forward primer NusG2a-F on saliva samples from 25 CRC patients and seven HCs (Table 7; Supplementary Materials, Table S3).

Subsequently, a total of 13 stool samples that were positively detected in previous experiments by all four L1-specific primer pairs were tested with the species-specific primers. The analysis revealed generally diverse *Fusobacterium* profiles across samples, with some samples showing co-colonisation by various L1 species. For example, sample 46 contained *F. animalis* clade 2 (5.2 × 10^4^ cells, 0.419%), *F. polymorphum* (2.9 × 10^3^ cells, 0.023%) and *F. vincentii* (2.5 × 10^4^ cells, 0.198%). In contrast, samples 45, 55, 70 and 73 contained only one species (*F. animalis* clade 1 in 70, *F. animalis* clade 2 in 45 and 55 or *F. polymorphum* in 73) (Supplementary Materials, Table S4).

Cross-reactivity was minimal; specifically, NusG-Fna2 as a reverse primer showed no non-specific amplification. Furthermore, reverse primers NusG-Fna1, NusG-Fnn, NusG-Fnv and NusG-Fnp exhibited a four- to five-log difference in sensitivity between target- and non-target (sub-)species.

### 2.9. Sanger Sequencing for Species-Specific Detection in Saliva Samples

To validate the specificity of the newly designed primers, Sanger sequencing was performed on the purified qPCR products of all samples that showed amplification. The resulting sequence types confirmed that all primers generally amplified the intended target species (See Clustered Heatmap under Discussion and Supplementary Materials, Table S6). Only 1 out of 12 samples sequenced from the NusG-Fnp (*F. polymorphum*) assay was misidentified as *F. vincentii* (labelled with *). The remaining eleven samples comprised four different *F. polymorphum-nusG*-sequence types, but none of them matched the sequence type found in the corresponding stool samples.

The five NusG-Fnv (*F. vincentii*) sequences all matched best with the same NCBI-reference (strain KCOM 3370), but differed from the *F. vincentii nusG*-sequence identified in the stool of the same patient.

Among the eight NusG-Fna2 (*F. animalis* clade 2) sequences, four different variances were found, none exactly matching those found in the stool samples.

All NusG-Fna1 (*F. animalis* clade 1) sequences were classified as the same variance (matching strain KCOM 3685), but distinct from the sequence identified in the stool samples.

Overall, the sequencing results support the high specificity of our newly designed species-specific primers.

### 2.10. Statistical Analysis of Species-Specific Primers

**Figure 3:**
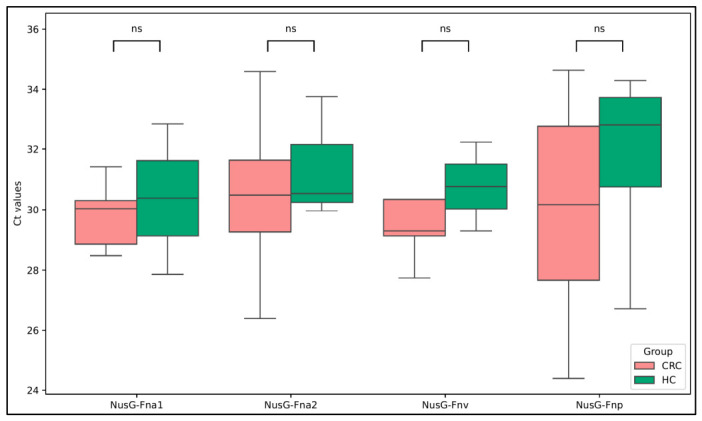
Boxplot representing the distribution of Ct values across four *Fusobacterium* species-specific primers in saliva samples from colorectal cancer (CRC) patients versus healthy controls (HCs). Lower Ct values indicate higher bacterial abundance. The *n*-values in the plot reflect samples with detectable amplification in qPCR from a total cohort of *n*_CRC_ = 25 and *n*_HC_ = 7. Central horizontal lines represent the median, boxes indicate the interquartile range (IQR), while whiskers extend to 1.5× IQR. Outliers were omitted for visual clarity. Statistical significance between groups was assessed using the Mann–Whitney U test (ns = not significant). Red = CRC, green = HC. Although not significant, a trend towards higher Ct and thus lower fusobacterial cell numbers in saliva of HC becomes obvious. Created with Seaborn (version 0.13.2)/Matplotlib (version 3.10.5) in Python (version 3.13.7).

Descriptive statistics, including mean, *SD*, median and *IQR*, were calculated for all qPCR data. For the saliva samples, all primer pairs showed lower mean and median values in the CRC group compared to the HC group (Figure 3).

The Mann–Whitney U test was performed to compare Ct values as well as absolute cell counts between CRC and HC groups in the saliva samples. After *FDR* correction, none of the primer pairs reached statistical significance (all *p_adj_* > 0.7) and failed to achieve a large effect size (all |*r*| < 0.3, *r*^2^ < 0.09).

To compare Ct values in paired saliva and stool samples, the Wilcoxon signed-rank test was performed, but for none of the primer pairs, statistical significance or a large effect size could be observed.

**Figure 4:**
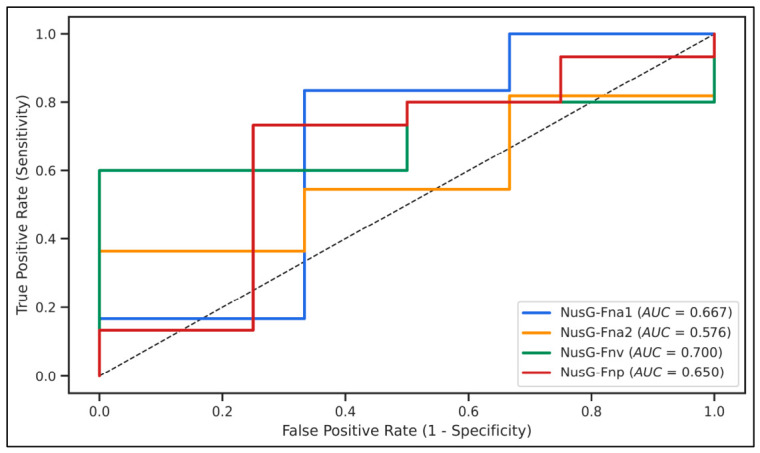
Receiver operating characteristic (*ROC*) analysis of four *Fusobacterium* species-specific *nusG*-directed qPCR assays in saliva samples. The curve illustrates the diagnostic performance of four different primer pairs in discriminating between colorectal cancer (CRC) patients and the healthy control (HC) group. The area under the curve (*AUC*) values for each primer pair are provided in the legend, representing the overall predictive accuracy, and the dashed diagonal line represents the performance of a random classifier (*AUC* = 0.500). Specificity and sensitivity levels were calculated across all potential Ct threshold values. Created with Seaborn (version 0.13.2)/Matplotlib (version 3.10.5) in Python (version 3.13.7).

Using *ROC* analysis, the diagnostic performance was assessed (Figure 4). The discriminatory potential was generally limited throughout all primer pairs, with NusG2a-F/NusG-Fnv achieving the highest *AUC* value at 0.700, with a sensitivity of 0.60 and a specificity of 1.0 at the optimal Ct cut-off of 29.29. The remaining primer pairs range in *AUC* values between 0.5 and 0.7, indicating low discriminatory power.

Spearman’s rank correlation was applied to evaluate the relationship between the paired saliva and stool samples. None of the species-specific PCR results depicted any statistically significant association. However, it must be taken into account that this part of our analyses is truly underpowered due to the small number of paired saliva and stool samples from HC (eight saliva samples were exhausted). The species level is thus too low to demonstrate significance, as the results tables include many “0” values.

## 3. Discussion

### 3.1. The Diagnostic Potential of Fusobacterium nusG-Directed PCR

While CRC screening methods significantly reduce CRC-specific mortality, their effectiveness remains dependent on high patient adherence [34]. Although colonoscopy is still considered the gold-standard technique, the initial patient acceptance is often higher for non-invasive procedures [35]. Patients are frequently deterred by fear of the invasive procedure itself, by the inconvenience of the bowel preparation/investigation [36], or the possible occurrence of complications [37]. While the faecal immunochemical test (FIT) serves as a non-invasive, highly specific diagnostic tool, it exhibits limited sensitivity, especially regarding advanced adenomas [38]. Furthermore, other non-malignant sources of bleeding, such as haemorrhoids, can lead to false-positive results [39]. This highlights the potential for additional non-invasive, accurate early diagnostic methods to support the existing screening procedures. In this context, the oral microbiota has been recognised as a promising source of biomarkers, as distinct microbial signatures can be observed in both oral and faecal samples of CRC patients [40]. Our results demonstrate that the *nusG* gene is a highly promising target for the development of primers to amplify and differentiate *Fusobacterium* species in clinical samples, as exemplified here using saliva and stool samples from CRC patients. Regarding primer quality, it is noteworthy that *nusG*-directed primers—similar to *rpoB*-targeted primers—contain up to five wobble positions in order to ensure coverage of all L1 Fusobacteria. However, the optimal *nusG*-directed primer pair combinations identified after an intensive screening process contained a maximum of one triple-wobble base (e.g., D: A/G/T or H: A/C/T), whereas previous *rpoB*-based assays contained one or even two triple-wobbles in both the forward and reverse primers [10]. This might explain the improved performance observed in our assays.

Our study successfully designed and applied four *Fusobacterium*-L1-specific and five *Fusobacterium* (sub-)species-specific qPCR assays in a clinical setting using 25 reference strains and a limited number of paired saliva and stool samples. The *nusG* gene has been previously recognised as a suitable target for *Fusobacterium* detection, as multiple studies have acknowledged its diagnostic potential [41,42,43]. However, they failed to differentiate between L1 species and merely defined the species as “*F. nucleatum*”, even though the specification was deemed necessary by recent findings [12,44]. The four L1 primer pairs were all capable of detecting *Fusobacterium* L1 in stool samples, with varying sensitivity and specificity. The five species-specific primers all reliably detected their respective target species in both niches, stool and saliva. Most importantly, the sequencing results confirmed the specificity and accuracy of the employed primer pairs. The chosen primer combinations—after a step-by-step selection process—demonstrated sufficient discriminatory power overall, reinforcing the well-established connection between *Fusobacterium* enrichment and colorectal tumours [15,16]. The best diagnostic performance was observed for NusG5-F/NusG6-R and NusG2a-F/NusG5-R with *AUC* values of 0.909 and 0.883, respectively. Their performance holds the chance to identify CRC patients during the early-onset stage 0 and 1 by excluding HC, a finding supported by the significant differences observed in the Mann–Whitney U test for these two primer pairs. Importantly, the sensitivity (82%) and specificity (90%) of the best-performing primer pair (NusG5-F/NusG6-R) demonstrate the potential to achieve performance metrics within the range of other established non-invasive procedures, such as the FIT (79%/94%), the faecal DNA test (92%/87%) or the SEPT9 test (68%/80%) [45]. While our findings are promising, they warrant further validation in larger and more ethnically diverse cohorts. It is important to emphasise that screening tools, such as colonoscopy and the FIT, are supported by extensive research and decades of clinical validation. Our objective was not to replace these well-established methods, but rather to provide a complementary solution, as a *nusG*-based molecular assay may address existing diagnostic gaps.

In summary, our data indicates that qPCR primers based on the *nusG* gene hold potential to be utilised as a promising, non-invasive component of early CRC diagnostics.

### 3.2. Methodological Optimisation

Regardless of the overall success, issues concerning assay sensitivity need to be discussed. While high sensitivity is generally preferable in a diagnostic context, our findings emphasise the importance of defining a clear physiological cut-off value to distinguish between transient bacteria (those swallowed or ingested) and active ectopic colonisation. The NusG1-F/NusG1-R example illustrates this particularly well, as the detection limit was as low as 10^3^ CFU/sample, yet the diagnostic accuracy (*AUC* of 0.642) was the lowest because differentiation between the CRC and HC group failed. This suggests that detecting *Fusobacterium* at low concentrations is undesirable, as it likely represents merely a transient physiological presence. In this context, a study by Segata et al. from 2012 described the average abundance of Fusobacteria in the stool of healthy individuals as 0.067% [46], but this includes mainly intestinal, non-L1 Fusobacteria. According to Connolly and Kelly 2025 [9], the L1-gut load in healthy individuals is age-dependently growing and might reach a maximum of 0.08% in the elderly group, whereas it is generally regarded as much higher in CRC (and other patients such as Crohn’s disease in males) [9]. In another study, a 132-fold increase in the abundance of *F. nucleatum* alone was observed with CRC compared to HCs [47]. Also, *F. nucleatum* was detected in 47.7% of stool samples from CRC patients, whereas it was present in only 7% of the HC group [48]. In conclusion, here, the definition of a distinct physiological threshold is needed to distinguish a CRC-associated gut microbiome from a healthy one.

### 3.3. Biological Significance

**Figure 5:**
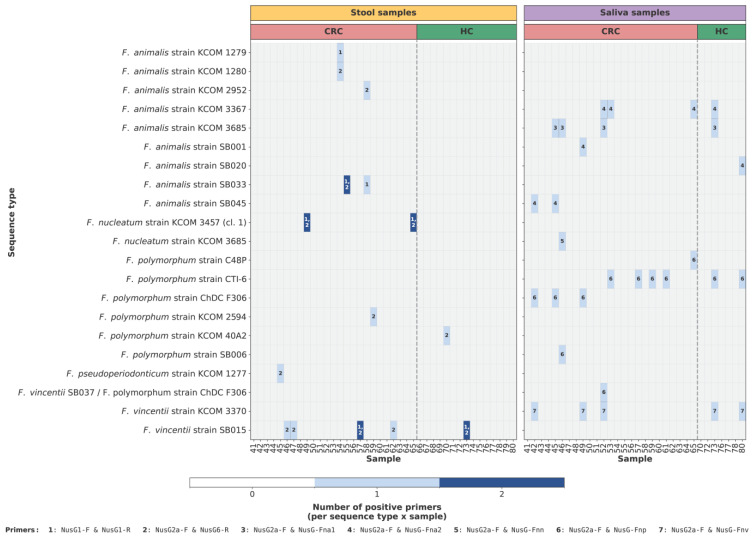
Clustered Heatmap of *Fusobacterium* sequence types across clinical samples. The heatmap displays all sequencing results for colorectal cancer (CRC, red) patients and healthy controls (HC, green) in stool (yellow) and saliva (purple) samples. Rows represent identified sequence types, while columns show individual samples. The colour scale indicates the frequency of a sequence type occurrence in the sample across seven distinct *nusG*-directed primer combinations (numbered 1 to 7), numbers within the cells correspond to the specific primer combinations listed. Created with Seaborn (version 0.13.2)/Matplotlib (version 3.10.5) in Python (version 3.13.7).

A key aim of this study was to compare the *Fusobacterium* composition in a microbiome context in paired stool and saliva samples from CRC patients and HC. The diverse microenvironments of the oral cavity and the gut—characterised by different microbial cell densities, water/mineral content, oxygen and nutrient availability, and pH levels—limit the direct comparability of these two sample types. This distinction was further supported by our sequencing results. No *Fusobacterium* (sub-)species exhibited the same sequence type in both stool and saliva samples from the same individual. This suggests that there is a separation between the oral and enteric niches, which contradicts the hypothesis that the enrichment of Fusobacteria in colorectal tumours is solely due to continuous passage from the oral cavity. However, these results diverge from those of Komiya et al., who did identify identical strains in paired oral and intestinal CRC samples [49]. This correlation was further supported by a 2023 study by Shimomura et al., which utilised targeting of the CRISPR-Cas region for strain-level detection [50]. Despite the methodological differences, including the potential for strain resolution, these conflicting findings can be reconciled by acknowledging that, while several strains must overlap in both niches, they undergo microevolution and selection during gastrointestinal passage. Consequently, the most abundant strains in the oral cavity and gut are not necessarily the same. This suggests that ectopic L1-*Fusobacterium* populations colonising the gut are highly specialised and possess specific selective advantages that enable them to survive the conditions within the gastrointestinal tract. Consequently, while diverse L1-*Fusobacterium* strains are constantly swallowed, only certain adapted strains can withstand the selective pressures of the gastrointestinal passage and establish themselves. This is a result of a higher repertoire of specific virulence factors such as Fap2 or FadA that facilitate adhesion on tumour tissues and enrichment in the tumour environment [11,20,21]. The hypothesis of a selection of specific strains is supported by recent findings by Zepeda-Rivera et al., exposing specifically *F. animalis* clade 2 as the dominant coloniser of CRC and connecting this association to the identification of distinct genetic factors enriched in these strains [12]. Moreover, *nusG*-sequence-type diversity among the distinct L1 species was a recurring motif and formed a consistent pattern across both sample types (Figure 5). *F. vincentii* and *F. animalis* clade 1 displayed low strain diversity, with only one single sequence identified in each respective sample type. Meanwhile, *F. polymorphum* and *F. animalis* clade 2 demonstrated greater variety, with two to four different sequence types found in each case. These contrasts indicate a distinct adaptation or competition behaviour of the diverse *Fusobacterium* species, which is in line with findings that suggest a clade-specific pathogenesis of Fusobacteria [51].

### 3.4. Limitations of the Study

When interpreting the results, several limitations of the present study must be addressed.

Foremost, the sample size and cohort characteristics reduce the significance of the data. The relatively small cohort of 25 CRC patients and 15 HC (with some saliva samples even exhausted) must be acknowledged. This limits the statistical power; therefore, the diagnostic performance observed here should be interpreted as preliminary rather than definitive. Furthermore, the cohort was restricted to a single geographical origin, namely, Iranian patients. It is important to consider that the composition of the oral and gut microbiome is highly influenced by regional dietary patterns, lifestyle factors and host genetics. Therefore, experiments with larger cohorts with patients from different, independent geographical origins are needed in future studies to verify the results.

Additionally, the extent of the conclusions is narrowed by the fact that not all CRC cases are connected to microbial dysbiosis inside the gastrointestinal tract. An estimated 10% are hereditary and therefore primarily attributable to genetic factors, namely due to specific mutations such as familial adenomatous polyposis or Lynch Syndrome [52]. These cases would likely not exhibit the characteristic difference in *Fusobacterium* abundance and would therefore not be detectable by our qPCR assays. It is evident that sporadic, microbially associated CRC subtypes represent the primary targets for fusobacterial *nusG*-directed—or, indeed, any microbiome-based—assays.

Finally, the methodology itself poses an inherent limitation, as qPCR cannot differentiate between DNA from viable/living and DNA from non-viable/dead cells or free DNA. Accordingly, the data does not depict the active *Fusobacterium* population, but rather the absolute biomass. Therefore, this method by itself cannot directly connect *Fusobacterium* load to disease progression, which is presumably driven by the secretion of specific virulence factors.

### 3.5. Future Perspectives

There are different approaches to further increase the assay’s sensitivity (above 82%) in future applications.

Firstly, lowering the annealing temperature is a standard strategy; however, this method must be carefully weighed against the risk of limited specificity, especially regarding cross-reactivity with non-L1 *Fusobacterium* species (L2–L9, see Supplementary Figure S1) or swallowed, transient oral fusobacterial cells.

Secondly, the difficulty of sensitivity could alternatively be addressed through the primer composition, namely the usage of inosine. Incorporating inosine, a universal base analogue capable of pairing with all four nucleotides, at degenerate positions may strengthen future assay designs. This may be particularly beneficial given that the high diversity of *Fusobacterium* strains requires including multiple degenerate bases in our current primer design. It would enhance the effective concentration of the functional primer by avoiding a dilution of the primer mixture, thereby increasing the sensitivity.

Thirdly, a Long-Taq polymerase system could address sensitivity issues resulting from longer fragments, such as NusG5-F/NusG1-R at 356 bp. The pairing performed well in endpoint PCR but was excluded from testing with patient samples due to low sensitivity in qPCR, likely attributable to the decreased processivity of standard Taq polymerase on longer amplicons. By pairing standard Taq with a high-fidelity enzyme, a Long-Taq system enhances the enzyme’s ability to execute synthesis, thereby accomplishing higher sensitivity on longer templates. This would also enable a multiplex assay combining shorter and longer amplicons.

Fourthly, rather than the two-primer-based qPCR method examined in this study, a Taqman format incorporating a third, internal Taqman probe could be employed to enhance specificity, if required.

Lastly, not for all *F. nucleatum* L1 members in humans, a species-specific PCR assay was developed. The main reason was that our sequencing results (see Supplementary Materials, Table S5) never detected *F. hwasookii* or *F. pseudoperiodonticum* in more than one case. However, an implication of these species in carcinogenesis cannot be excluded and might even be understudied.

Our findings indicate that stool is a suitable medium for detecting *Fusobacterium* abundance differences between CRC patients and HCs using *nusG*-based primers. In contrast, no significant difference was detected in saliva samples. While this finding is consistent with a recent systematic review and meta-analysis, stating the lack of a correlation between CRC and abundance of *F. nucleatum* in saliva [53], it is critical to note our limited salivary sample size (*n* = 7 for HC). Consequently, our study was not adequately powered to conclude that saliva may have limited discriminatory power to identify gut dysbiosis. As previously discussed, the fusobacterial fraction of the oral environment does not directly reflect the gut abundance of Fusobacteria. The discovery of more similar quantities in CRC and healthy saliva compared to stool could be especially interesting, as a current hypothesis suggests that, while highly abundant in stool, Fusobacteria might in fact be reduced in the saliva of CRC patients [54]. This reduction could be caused by a systematic inflammatory response originating from the colorectal tumour, which could subsequently affect the oral microenvironment by modulating the “oral–gut–circulatory axis” [55] and by the release of specific pro-inflammatory cytokines such as IL-6 or TNF-α [56]. This biological hypothesis warrants further investigation in larger, sufficiently powered studies and could provide new research opportunities if confirmed.

Beyond CRC, the methodology and primers developed in this study may offer broader utility in investigating *Fusobacterium*-associated malignancies. Primarily, *F. nucleatum* was among 10 bacterial species significantly enriched in oral microbiota samples of oral squamous cell carcinoma (OSCC) patients compared to healthy individuals, according to a 2020 study [57]. OSCC is responsible for approximately 90% of all oral malignancies and, similar to CRC, will see rising incidences in the next decades, associated with an increased mortality [58]. Despite the limited salivary sample size, our *nusG*-based primers represent a potential tool for future research into OSCC in larger-scale studies, as they demonstrated the ability to specifically detect relevant L1 (sub-)species in saliva samples.

Additionally, *F. nucleatum* was also found to be highly abundant in pancreatic and oesophageal cancer tissues, where its presence was associated with a poor patient prognosis, leading to a shorter survival [59,60]. Furthermore, *F. nucleatum* was also significantly enriched in breast cancer tissues [61], where it, similarly to CRC, reaches the Gal-GalNAc-rich tumour cells via the Fap2 lectin and promotes tumour progression as well as metastasis [62].

Its role in oral health needs to be emphasised: *F. nucleatum* is an established key pathogen in periodontal disease [5]. Consequently, chronic periodontitis is a proven, independent risk factor for all major *Fusobacterium*-associated malignancies, including CRC, OSCC and oesophageal as well as pancreatic tumours [63,64]. This linkage highlights the need for extensive research on the oral–gut–circulatory axis and suggests that effective preventive and therapeutic measures for periodontitis through regular screening and intensive oral hygiene could potentially reduce systemic cancer risks.

## 4. Materials and Methods

**Figure 6:**
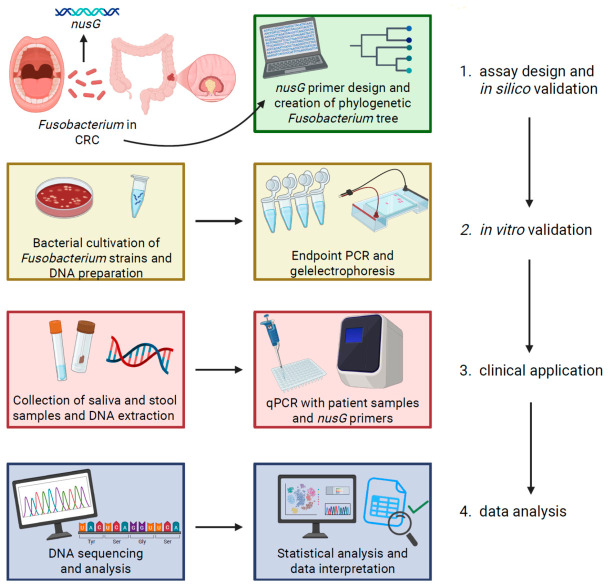
Graphical abstract of study methodology. Created in BioRender. Becker, J. (2026), BioRender.com/gzbm0lx.

A comprehensive overview of the methods used in this study is presented below in Figure 6. The detailed process will be described in the following sections.

### 4.1. Phylogenetic Analysis

To establish a robust evolutionary framework for primer design, a phylogenetic tree of Fusobacteria was constructed based on the complete sequences of the *nusG* and *rpoB* genes using MEGA12 (Molecular Evolutionary Genetics Analysis, version 12.1.2, build 250720) [65]. Best-fitting nucleotide substitution models were selected based on the lowest Bayesian information criterion (BIC) scores. Evolutionary history was inferred using the maximum likelihood method. For the heuristic search, the neighbour-joining method was applied. Topology reliability was tested with 5000 bootstrap replicates using *Ilyobacter polytropus* as the outgroup. Further visualisation and layout were refined in Inkscape (version 1.4.2). Further details and specific model parameters are provided in the legend of Figure S1 in the Supplementary Materials.

### 4.2. Primer Design

PCR primers were designed to specifically distinguish cancer-associated L1 strains from other Fusobacteria. The primers targeting the *nusG* gene of Fusobacteria were designed using the MEGA11 software (Molecular Evolutionary Genetics Analysis, version 11.0.13, build 220624) [66]. Initially, the gene sequences were retrieved via NCBI BLAST (version 2.17.0) using the type strain *F. nucleatum* subsp. *nucleatum* (ATCC 25586) as the initial query. The acquired sequences from 255 different *Fusobacterium* strains then underwent sequence alignment and comparison via MEGA11. The aim was to find highly conserved sequences within the cancer-associated L1 Fusobacteria that showed distinct variation compared to non-L1 strains due to single-nucleotide polymorphisms (SNPs), especially at the 3′ primer end for optimal performance. Finally, internally conserved regions of L1 strains with the most informative SNPs were selected for primer design. A varying number of degenerate (wobble) bases had to be incorporated due to intra-lineage sequence variability within the chosen regions. The designed primers possess a GC content below 40% in contrast to the recommended 40–60%, a matter that was tolerated to accomplish a higher specificity and to prevent cross-reactivity (Table 8).

For (sub-)species-specific detection, the newly designed L1-directed forward primer NusG2a-F was then paired with five reverse primers, each targeting a specific *Fusobacterium* (sub-)species. These reverse primers were designed from conserved regions of the respective (sub-)species following the same procedure as for the L1 primer design and included degenerate bases where necessary to ensure amplification specificity (Table 8).

### 4.3. Bacterial Strains and Growth Conditions

To provide validated material for DNA testing, a number of 25 *Fusobacterium* strains (Supplementary Materials, Table S7) were grown on TSASB plates (Tryptic Soy Agar with Sheep Blood Medium; Thermo Fisher Scientific Inc.™; Waltham, MA, USA) at 37 °C under anaerobic conditions via GENbox anaero generators (bioMérieux S.A.; Marcy-l’Étoile, France) for 2–5 days, depending on the growth activity.

### 4.4. DNA Preparation

#### 4.4.1. DNA Preparation of *Fusobacterium* Strains

To ensure reproducible results, high-purity genomic DNA was extracted from bacterial cultures, stool and saliva samples using standardised, kit-based protocols. Initially, colonies were picked from the plates and resuspended in 250 µL of sterile distilled water. DNA extraction was then performed according to the QIAamp^®^ DNA Mini Kit DNA Purification Spin Protocol (Qiagen N.V.; Venlo, The Netherlands). Following the extraction, a sufficient concentration and purity of the DNA were ensured by applying the NanoVue Plus Spectrophotometer (GE Healthcare Europe GmbH; Freiburg, Germany). The extracted DNA was stored frozen in aliquots to ensure reproducible measurements over time.

#### 4.4.2. Study Population and DNA Preparation of Stool and Saliva Samples

The present study was a case–control analysis, with all samples procured from patients referred for standard screening colonoscopy between February 2020 and January 2022 at Taleghani Hospital in Tehran, Iran. Enrolled participants, aged ≥18 years, were recruited from patients referred to Taleghani Hospital from different regions of Iran. A total of 40 stool and saliva samples were collected, comprising 15 HC and 25 CRC cases, prior to colonoscopy procedures. Participant status (CRC vs. HC) was determined by colonoscopy and TNM-stage (“early”, 0–1), subsequently confirmed by histopathological examination. Patients exhibiting symptoms such as rectal bleeding, changes in bowel habits, anaemia, and abdominal pain were included in the study, while exclusion criteria comprised individuals: (1) who had taken antibiotics within the past three months; (2) who had regularly used probiotics within the past three months; (3) who had undergone invasive medical interventions within the same period; (4) with a previous history of CRC, inflammatory or infectious intestinal diseases; (5) with gastrointestinal disorders like inflammatory bowel disease, irritable bowel syndrome, liver disorders, or non-alcoholic fatty liver disease; and (6) categorised as high-risk for CRC, such as those with familial adenomatous polyposis or other hereditary cancer syndromes. Demographic and clinical data, including age, sex, smoking status, alcohol consumption, and dietary patterns, were collected through standardised questionnaires (Supplementary Materials, Table S8). HC participants were chosen based on normal colonoscopy results and a negative personal and family history of gastrointestinal diseases. Patients were approached during their initial hospital visit to explain the research protocol and obtain informed consent.

Samples were collected in the morning (between approximately 8:00 and 12:00) under fasting conditions, with participants instructed not to eat or drink for at least 2 h prior to sampling. Importantly, there was no requirement for oral hygiene before collection, so participants did not necessarily brush their teeth, possibly increasing the salivary cell load. Saliva was collected as unstimulated whole saliva. Participants were given time (approximately 5–10 min) to passively accumulate saliva and spit into the collection tube. All collected samples were kept frozen at −20 °C minimum, thawed when needed, vortexed and centrifuged briefly, mainly in order to homogenise the sample but possibly further increasing the salivary cell load.

The DNA of 0.16 g (average wet weight) of stool samples from 25 CRC patients and 15 HCs was extracted using the QIAamp Fast DNA Stool Mini Kit (Qiagen N.V.; Venlo, The Netherlands) according to the manufacturer’s instructions. For oral samples, DNA of 0.1 mL (where available) of unstimulated saliva was isolated with the ZymoBIOMICS DNA Mini Kit (Zymo Research Corporation; Irvine, CA, USA), also following the manufacturer’s protocols. Of the initial 15 HC-saliva samples, 8 were unavailable for this primer-evaluation study due to being used in parallel studies addressing other topics.

### 4.5. Preparation of a Standard Curve for Quantitative Real-Time PCR

The qPCR was primarily established to selectively quantify the L1 *Fusobacterium* cells, which are cancer-associated, from the non-L1 species. Therefore, two strains were initially chosen: *F. animalis* clade 2 (OMI 1357) as a positive control for strains of L1 and *F. varium* (ATCC 8501)*,* belonging to lineage 5, as a negative control for L1 strains. The strains were first cultured anaerobically for several days at 37 °C on TSASB agar plates. They were then checked for purity and transferred to BHI liquid medium (Thermo Fisher Scientific Inc.™). The liquid culture was also cultivated for 4 to 6 days at 37 °C under anaerobic conditions and afterwards centrifuged at 8000 rpm for 4 min (centrifuge 5424; Eppendorf SE; Hamburg, Germany). The pellet was then prepared for a serial dilution series, which was utilised for (i) direct cell counting under the microscope using the “Neubauer Chamber” (Brand GmbH + Co. KG; Wertheim, Germany) as well as (ii) CFU counting from TSASB agar plates after sufficiently long anaerobic cultivation. Finally, the standard curve was generated by correlating the Ct values with the measured cell counts. For the qPCR using our five species-specific reverse primers, additional standard curves were prepared to include the respective (sub-)species. Accordingly, four strains, including *F. animalis* clade 1 (OMI 1486), *F. nucleatum* (ATCC 25586), *F. polymorphum* (OMI 1593) and *F. vincentii* (OMI 1416) were cultivated and processed following the same protocol as described above.

### 4.6. PCR

#### 4.6.1. Endpoint PCR and Agarose Gel Electrophoresis

Firstly, a mastermix was created consisting of 40 µL nuclease-free water, 5 µL 10× PCR buffer with MgCl_2_ (Roche Diagnostics GmbH; Mannheim, Germany), 2.5 µL dNTPs (Roche Diagnostics GmbH), 0.5 µL each of forward- and reverse Primer (synthesised by TIB Molbiol Syntheselabor GmbH; Berlin, Germany) and 0.5 µL Taq Polymerase (Roche Diagnostics GmbH). A volume of 1 µL of DNA was added and cycling was conducted via the PCR Express Thermal Cycler (Thermo Hybaid, subsequently Thermo Fisher Scientific; Waltham, MA, USA) using the following parameters: initial denaturation at 94 °C for 2 min; 35 cycles with denaturation at 94 °C for 30 s, annealing at 52–58.5 °C (based on data from Table 1), extension at 72 °C for 1 min, and final extension at 72 °C for 5 min. Following the cycling, gel electrophoresis was performed to verify the presence and length of the expected amplified product. Therefore, agarose gels ranging from 1.5% to 3%, depending on the fragment size, were prepared in 1× TAE buffer (40 mM Tris-acetate, 1 mM EDTA, pH 8.5). For this, 0.45 to 0.9 g of agarose (Merck KGaA, Life Science; Darmstadt, Germany) was added to 30 mL of 1× TAE buffer and dissolved by heating. After cooling, 1.5 µL of Midori Green Advance (NIPPON Genetics EUROPE GmbH; Düren, Germany) was added and mixed thoroughly. After pouring the mixture into the gel chamber, placing the gel comb and allowing the gel to polymerise, 10 μL of GeneRuler 100 bp DNA Ladder (Thermo Fisher Scientific Inc.™) was loaded into a single well. Subsequently, 10 μL of each sample premixed with BlueMarker (40% sucrose, 0.1% bromophenol blue) was loaded into individual wells. The gel electrophoresis was then conducted using a voltage of 100 V and an amperage of 250 mA for 30 to 40 min (Consort EV243 Electrophoresis Power Supply; Consort nv; Turnhout, Belgium), depending on the expected length of the fragment. The resulting bands in the gel were then visualised under UV light (GelDoc Go Gel Imaging System; Bio-Rad Laboratories, Inc.; Hercules, CA, USA).

#### 4.6.2. Quantitative Real-Time PCR (qPCR) Targeting *Fusobacterium*

Quantitative real-time PCR was performed in a total reaction volume of 20 µL consisting of 10 µL SYBR™ Green Universal Master Mix (Thermo Fisher Scientific Inc.™), 5–8 µL of nuclease-free water, 0.5 µL each of forward primer and reverse primer (TIB Molbiol) and 1–4 µL of DNA. All qPCR reactions were conducted using the following parameters: initial denaturation at 95 °C for 10 min; 35 cycles with denaturation at 95 °C for 15 s, annealing at 50–60 °C (based on data from Table 1 and Table 6) for 15 s, extension at 72 °C for 1 min; and final extension at 72 °C for 5 min (QuantStudio 3 Real-Time PCR System; Applied Biosystems/Thermo Fisher Scientific, Waltham, MA, USA).

Each reaction was carried out in triplicate whenever possible, and non-template controls were always included to monitor potential contamination. To ensure specific amplification, the melting curve plot was monitored after each reaction.

### 4.7. PCR Clean-Up and Sequencing

To confirm the identity of the amplified products, all PCR fragments were purified and validated via Sanger sequencing. The samples were purified using the NucleoSpin Gel and PCR Clean-up Kit according to the manufacturer’s instructions (Macherey-Nagel GmbH & Co. KG; Düren, Germany). Following that, the DNA concentration was measured using the NanoVue Plus Spectrophotometer. The samples were then stored at 4 °C up to 7 days prior to sequencing, which was conducted by Eurofins Genomics (Ebersberg, Germany).

### 4.8. Calculation of Absolute Cell Count by Universal qPCR

To quantify the total bacterial load in patient samples and to calculate the relative abundance of all L1 Fusobacteria as well as individual *Fusobacterium* (sub-)species within the overall bacterial community, a universal qPCR targeting the 16S-gene was performed with the primers 331-F and 797-R (for sequences see Table 8) at a Ta of 51 °C in 35 cycles. Each reaction was carried out in triplicate. The obtained Ct values were then utilised to calculate absolute cell counts per µL DNA extract and in 0.1 g of stool sample or 0.1 mL of saliva concentrate from CRC patients and HCs (Supplementary Materials, Table S1).

### 4.9. Statistical Analysis

All statistical analyses were performed in Python (version 3.13.7; Python Software Foundation; https://www.python.org) on macOS using established scientific libraries and tools pandas (version 2.3.1), NumPy (version 2.3.2), SciPy (version 1.16.2), statsmodels (version 0.14.5), Matplotlib (version 3.10.5), and Seaborn (version 0.13.2).

Mean and *SD*, as well as median and *IQR*, were provided for all qPCR data.

Since the qPCR data followed a non-normal distribution, all comparative analyses were carried out using non-parametric tests.

The Mann–Whitney U test was used for independent group comparisons (CRC vs. HC) for all primer pairs, while the Wilcoxon signed-rank test was applied for paired stool and saliva analysis for the species-specific primers.

To maintain a low *FDR*, all the obtained *p*-values were then adjusted with the Benjamini–Hochberg procedure; *p*-values below 0.05 were deemed statistically significant.

The effect size *r* was calculated to quantify the magnitude of the detected differences; in this regard, *r*^2^-values ≥ 0.25 (*r* values ≥ 0.5 or ≤−0.5, |*r*| ≥ 0.5) were considered a large effect.

To evaluate the clinical utility of our primers, we performed *ROC* analysis, using the *AUC* and Youden’s Index (*J*) to determine optimal diagnostic cut-offs.

To examine the relationship between saliva and stool samples, Spearman’s rank correlation analysis was used to calculate both the Spearman’s correlation coefficient (ρ) and the corresponding *p*-values.

## 5. Conclusions

During a time that calls for non-invasive, reliable diagnostic tools in the context of CRC diagnostics, our study—albeit in need of improvement and of limited significance—successfully demonstrates the high potential of *nusG*-targeted PCR primers. Specifically, the primer combinations NusG5-F/NusG6-R and NusG2a-F/NusG5-R were able to detect a statistically significant difference in *Fusobacterium* abundance between CRC patients and HCs, demonstrating promising diagnostic accuracy as validated by *ROC* analysis. While our experimental findings confirm that stool is a suitable medium for CRC screening, the inconclusive results regarding the saliva samples are likely attributable to the limited sub-cohort size (*n* = 7 for HCs). Consequently, the extent to which the saliva microbiome in general, or its fusobacterial fraction in particular, can reflect gut dysbiosis requires further investigation. Given the limited total cohort size and geographical origin of our study population, future research in larger, ethnically diverse cohorts is critical to validate the clinical applicability.

Future approaches may consider methodological optimisation with regard to the assay sensitivity via the incorporation of inosine bases, the usage of a Long-Taq system or transformation into an even more specific Taqman format.

Further research is required on the specific *Fusobacterium* species or pathotypes that successfully colonise the gut, along with their distinct virulence factors. In addition, the role of *Fusobacterium* in other cancers and the potential use of *nusG*-targeted PCR primers in that context must be investigated. The potential of accurate, low-barrier, cost-effective screening tests that target the (partial) microbiome should not remain underutilised.

## Figures and Tables

**Table 1:** Characteristics of all *Fusobacterium* L1 *nusG*-directed primers selected after in silico analyses.

Primer	Orientation	Primer Length [bp]	Number of Wobble Bases	GC Content [%]	Annealing Temperature [°C]
NusG1a-F	forward	24	8	29.2	55.8
NusG1-F	forward	24	4	33.3	52.3
NusG2a-F	forward	27	2	22.2	54.9
NusG5-F	forward	29	4	34.5	62.7
NusG1-R	reverse	24	4	33.3	51.8
NusG5-R	reverse	31	4	35.5	63.0
NusG6-R	reverse	26	5	34.6	48.4
NusG7-R	reverse	25	4	32.0	52.3

**Table 2:** Overview of *Fusobacterium* L1 primer pairs and their corresponding amplicon lengths, NusG domain and start/stop base position. For more information, see Supplementary Materials, Figure S2.

Primer Combination	Amplicon Length [bp]	NusG Domain	Start/Stop Base Position [bp]
NusG1a-F/NusG1-R	111	KOW	438/548
NusG1-F/NusG1-R	111	KOW	438/548
NusG2a-F/NusG5-R	173	NGN	48/220
NusG2a-F/NusG6-R	232	NGN	48/279
NusG2a-F/NusG7-R	313	NGN	48/360
NusG5-F/NusG1-R	356	NGN and KOW	193/548
NusG5-F/NusG6-R	87	NGN	193/279
NusG5-F/NusG7-R	168	NGN	193/360

**Table 3:** Results of endpoint PCR accuracy applying eight *Fusobacterium* L1 primer pairs with reference strain DNA.

Primer Combination	Positive Control	Negative Control *F. varium*	Consequence
NusG1a-F/NusG1-R	Weak bands	No amplification	Further explored in qPCR
NusG1-F/NusG1-R	Weak bands	No amplification	Further explored in qPCR
NusG2a-F/NusG5-R	Weak bands	No amplification	Further explored in qPCR
NusG2a-F/NusG6-R	Strong bands	No amplification	Optimal
NusG2a-F/NusG7-R	Strong bands	Strong bands	Excluded
NusG5-F/NusG1-R	Strong bands	No amplification	Optimal
NusG5-F/NusG6-R	Weak bands	No amplification	Further explored in qPCR
NusG5-F/NusG7-R	Strong bands	Weak bands	Needs optimisation during qPCR

**Table 4:** Results of qPCR performance analysis of *Fusobacterium* L1 primer pairs on DNA standards of reference strains. Sensitivity was considered “sufficient” if *nusG*-gene equivalents of ≥10^4^ colony-forming units (CFU) of *Fusobacterium* per µL DNA were detected, as this is about the number found during true intestinal colonisation (corresponding to ≥0.08% of stool bacteria [9]).

Primer Combination	Mean Ct of 10^8^ CFU *	Detection Limit (*nusG* Gene Copy Numbers)	Sufficiently High Sensitivity
NusG1a-F/NusG1-R	25.38	≥10^6^	No
NusG1-F/NusG1-R	16.44	≥10^3^	Yes
NusG2a-F/NusG5-R	17.15	≥10^4^	Yes
NusG2a-F/NusG6-R	17.89	≥10^4^	Yes
NusG5-F/NusG1-R	21.83	≥10^5^	No
NusG5-F/NusG6-R	17.70	≥10^4^	Yes
NusG5-F/NusG7-R	20.58	≥10^5^	No

* Colony-forming units or *nusG*-gene equivalents, respectively, in standard: higher values mean lower PCR performance and make different primer combinations comparable.

**Table 5:** Sensitivity of *Fusobacterium* L1 qPCR analysis after applying on all 40 stool samples.

Primer Combination	Mean Ct of 10^8^ CFU	Detection Limit (CFU *)
NusG1-F/NusG1-R	15.63	≥10^3^
NusG2a-F/NusG5-R	16.71	≥10^4^
NusG2a-F/NusG6-R	17.26	≥10^4^
NusG5-F/NusG6-R	16.10	≥10^4^

* Colony-forming units or *nusG*-gene equivalents, respectively, in 1 µL DNA extract of stool.

**Table 6:** Characteristics of the *Fusobacterium* species-specific *nusG*-directed primers.

Primer	GC Content [%]	Annealing Temperature [°C]	Amplicon Length with NusG2a-F [bp]
NusG-Fna1	42.9	54.7	159
NusG-Fna2	38.1	51.5	126
NusG-Fnn	38.1	47.2	144
NusG-Fnp	47.6	53.0	240
NusG-Fnv	33.3	42.4	219

**Table 7:** Results of qPCR assays with *Fusobacterium* species-specific primers.

Reverse Primer	Ct of 10^8^ CFU	Detection Limit (CFU *)	CRC Salivary Samples (*n* = 25) Positive	HC Salivary Samples (*n* = 7) Positive
NusG-Fna1	14.10	≥10^2^	6	3
NusG-Fna2	15.12	≥10^4^	10	4
NusG-Fnn	16.80	≥10^4^	1	0
NusG-Fnp	14.97	≥10^3^	15	4
NusG-Fnv	16.82	≥10^4^	5	2

* Colony-forming units or *nusG*-gene equivalents, respectively, in 1 µL DNA extract of saliva. PCR in combination with the universal L1 forward primer NusG2a-F.

**Table 8:** Sequences of primers used in endpoint and qPCR in this study.

Name	Sequence 5′-3′	Orientation	Target Species/Cluster
NusG1a-F *	TTG RYT TTR CWG ARG GRG AYT WTG	fw	NusG KOW domain of all L1 species by exclusion of others
NusG1-F	TTG ACT TTR CWG ARG GAG AYT ATG	fw	
NusG1-R	CAA CCA WYA CTT TAR CTC TRC CAT	rv	
NusG2a-F	TGG ATA TGA AAA AAA RGT RAA AAC AGA	fw	NusG NGN of all L1 species by exclusion of others
NusG5-F	TTY CCW GCD TAT GTY ATG CTT GAA ATG GA	fw	
NusG5-R	CCA TTT CAA GCA TRA CAT AHG CWG GRA AAA G	rv	
NusG6-R	CCA YAC AYS AGG RTC TAC TTT ATA RC	rv	
NusG7-R	TAC YTC KTC HTC TTC CAT AGG AAT W	rv	
			NusG NGN of
NusG-Fna1	ACA TAT GCT GGG AAA AGC TTC	rv	*F. animalis* clade 1
NusG-Fna2	TTA GGT TTC CCT CTA ACA ATC	rv	*F. animalis* clade 2
NusG-Fnn	AGC TTT CTA TAG ACC TTC TTG	rv	*F. nucleatum*
NusG-Fnp	ACT TCA TAC CAW ACA CGA GGG	rv	*F. polymorphum*
NusG-Fnv	TCT ACT TTA TAG CTT ATA CCC	rv	*F. vincentii*
331-F	TCC TAC GGG AGG CAG CAG T	fw	16S rRNA gene of all bacteria
797-R	GGA CTA CCA GGG TAT CTA ATC CTG TT	rv	

* NusG1a-F is a variation of NusG1-F that includes *F. pseudoperiodonticum* by incorporation of three additional wobble bases.

## Data Availability

The original contributions presented in this study are included in the article/Supplementary Materials. Further inquiries can be directed to the corresponding author (G.C.).

## Supplementary Materials

The following supporting information can be downloaded at: https://www.mdpi.com/article/10.3390/ijms27114865/s1.

## Abbreviations

The following abbreviations are used in this manuscript:

*AUC*	area under the curve
CFU	colony-forming units
CRC	colorectal cancer
*FDR*	false discovery rate
FIT	faecal immunochemical test
FnL1	Human oral *Fusobacterium* species of lineage 1
HC	healthy control
*IQR*	interquartile range
KOW	NusG C-terminal domain
L1	lineage 1, *Fusobacterium nucleatum* complex
NGN	NusG N-terminal domain
NusG	N-utilisation substance G
OSCC	oral squamous cell carcinoma
PCR	polymerase chain reaction
qPCR	quantitative real-time polymerase chain reaction
RNAP	RNA polymerase
*ROC*	receiver operating characteristic
SD	standard deviation
SNP	single-nucleotide polymorphisms
