# Supplementary material for: Quantification of Ectopic Fusobacterium Colonisation in Colorectal Cancer Using a Newly Developed nusG-Directed PCR Method
Source: Int J Mol Sci. 2026 May 28;27(11):4865. doi: 10.3390/ijms27114865 (PMC13257272; doi:10.3390/ijms27114865)
Supplement: Supplementary file 1 [file ijms-27-04865-s001.zip › ijms-4251870-supplementary.pdf]

International Journal of *Molecular Sciences*

MDPI

Supplementary Materials

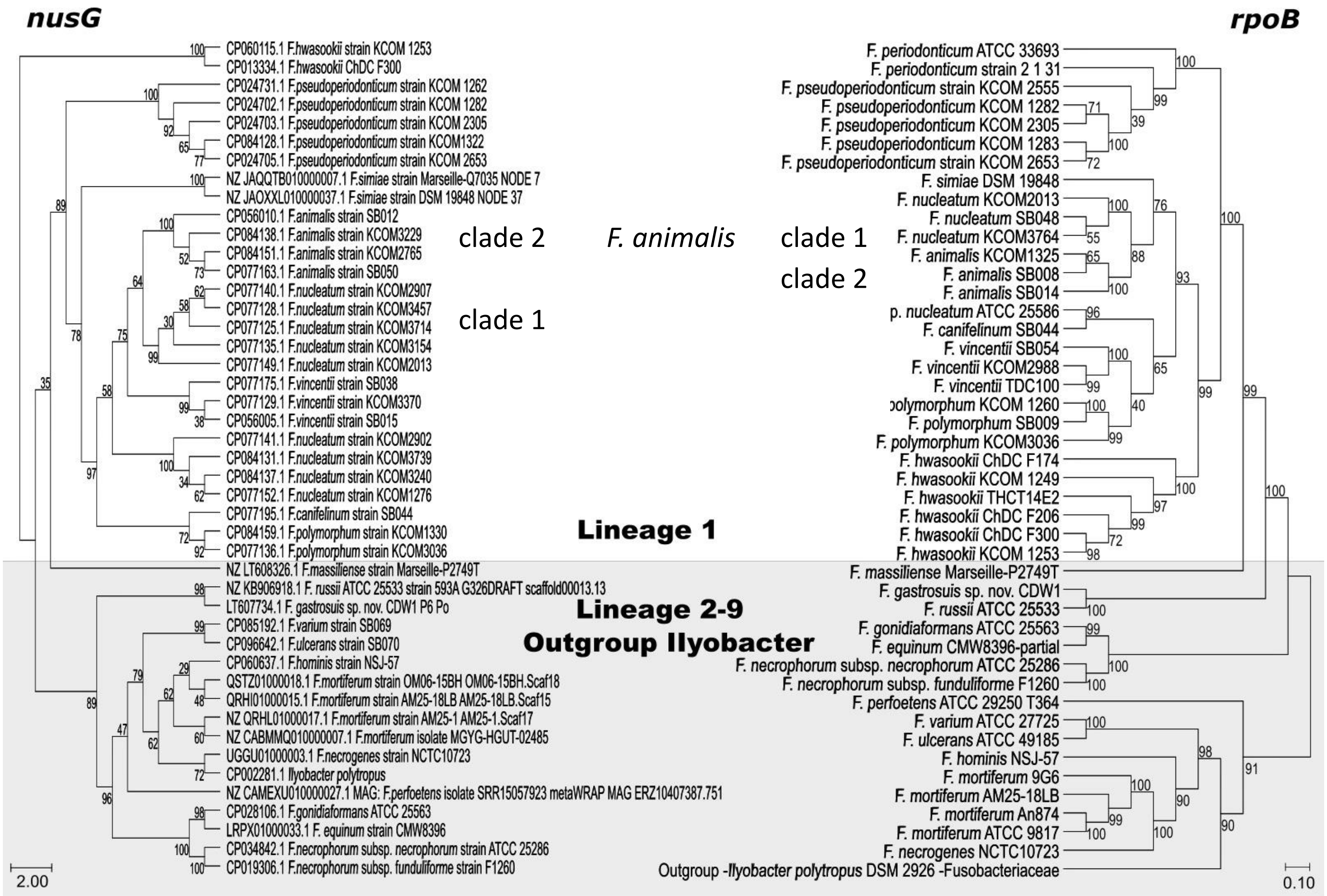

Figure S1: Phylogenetic tree of Fusobacteria of oral (L1) and non-oral (> L1) origin based on complete *nusG* and *rpoB* gene sequences. The closely related Fusobacteriaceae-species *Ilyobacter polytropus* was used as the outgroup. Evolutionary history was inferred by using the maximum likelihood method. For *rpoB*, the GTR+G+I (general time reversible + gamma distribution + proportion of invariant sites) model was applied; for *nusG*, the T92+G+I (Tamura 3-parameter + gamma distribution + invariant sites) model was used. The best fitting nucleotide substitution models were determined using the "Find Best DNA/Protein Models (ML)" function in MEGA12. Model selection was based on the lowest Bayesian information criterion (*BIC*) scores, and the top-ranked models in the MEGA output were applied. For the heuristic search, the neighbour-joining tree method was selected. Based on 5000 bootstrap replicates, the percentage of the trees in which the associated taxa clustered together is shown next to the branches as bootstrap values (%). The tree is drawn to scale, with branch lengths representing the number of substitutions per site. For visualization, the topology only mode was used. The trees were exported and edited in Inkscape (version 1.4.2), where both gene trees were arranged side by side in a single figure. Bootstrap values and branch length scale bars were transferred from the original ML trees without altering the topology or branch length information. Evolutionary analyses were conducted in MEGA12.

https://doi.org/10.3390/ijms27114865

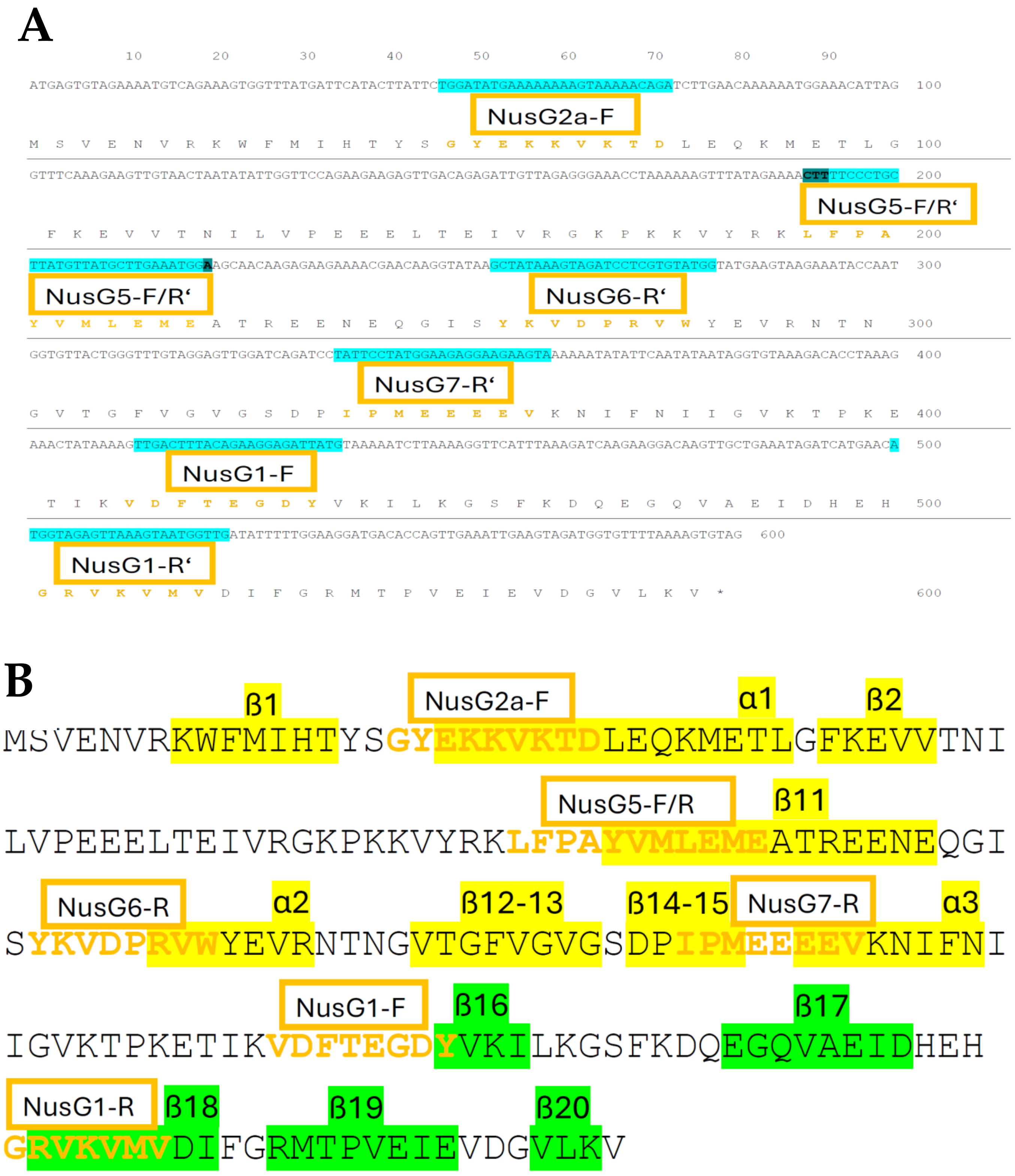

Figure S2: NusG-directed primer sites shown for the Clade 2- reference strain *Fusobacterium animalis* KCOM 1325 (NusG-protein ALF22583.1). **A)** Primer names indicate the specific *nusG*-gene annealing positions (AP, blue) of the respective L1 primers designed in this study. Although NusG5-F and NusG5-R originate from the same template site, their terminus (labelled in darker colour) differ slightly to optimise annealing. The amino-acid sequence corresponding to AP is highlighted in orange. **B**) NusG-protein sequence, AP of primers and corresponding structural elements of the NusG N-terminal domain (NGN, highlighted in yellow) and the C-terminal domain (KOW, highlighted in green) built by α-helices (α1-α3) and β-strands (β1-β20, with strands β3-β10 missing) compared to the reference aaeNusG of *Aquifex aeolicus* (Steiner et al. EMBO J. 2002 Sep 2;21[17]:4641-53).

Table S1: Total bacterial load* in patient-derived faecal and salivary samples determined by universal 16S rRNA qPCR.

Sample	Group	Total faecal bacterial load per µL DNA extract	Total faecal bacterial load per 0.1 g stool	Total salivary bacterial load per µL DNA extract	Total salivary bacterial load per 0.1 mL saliva
41	CRC	2.45E+07	4.90E+09	9.22E+03	1.48E+06
42	CRC	1.48E+07	2.96E+09	2.10E+07	4.20E+09
43	CRC	3.05E+06	6.10E+08	1.24E+06	1.98E+08
44	CRC	1.31E+07	2.62E+09	1.80E+07	2.88E+09
45	CRC	2.53E+07	5.06E+09	7.62E+06	1.52E+09
46	CRC	1.24E+07	2.48E+09	6.21E+07	9.94E+09
47	CRC	8.45E+07	1.69E+10	4.60E+05	7.59E+07
48	CRC	1.17E+05	2.34E+07	1.60E+06	2.56E+08
49	CRC	6.61E+07	1.32E+10	4.49E+06	8.98E+08
50	CRC	1.23E+07	2.46E+09	2.00E+06	4.00E+08
51	CRC	5.93E+05	1.19E+08	4.91E+07	7.86E+09
52	CRC	8.84E+04	1.77E+07	2.26E+07	3.37E+09
53	CRC	4.20E+07	8.40E+09	1.55E+07	2.56E+09
54	CRC	3.11E+07	6.22E+09	8.32E+06	1.66E+09
55	CRC	4.06E+07	8.12E+09	6.06E+06	1.21E+09
56	CRC	6.45E+07	1.29E+10	1.27E+07	2.54E+09
57	CRC	3.18E+07	6.36E+09	4.59E+07	7.57E+09
58	CRC	1.50E+05	3.00E+07	1.54E+06	3.08E+08
59	CRC	1.37E+08	2.74E+10	2.38E+07	3.93E+09
60	CRC	6.60E+06	1.32E+09	1.03E+07	1.65E+09
61	CRC	1.94E+07	3.88E+09	1.95E+07	3.22E+09
62	CRC	8.04E+07	1.61E+10	3.39E+06	5.42E+08
63	CRC	4.13E+06	8.26E+08	1.37E+07	2.19E+09
64	CRC	1.68E+07	3.36E+09	5.60E+04	1.12E+07
65	CRC	7.21E+06	1.44E+09	6.94E+05	1.15E+08
66	HC	7.46E+06	1.49E+09	n.d.	n.d.
67	HC	1.59E+07	3.18E+09	n.d.	n.d.
68	HC	2.62E+07	5.24E+09	n.d.	n.d.
69	HC	2.30E+07	4.60E+09	n.d.	n.d.
70	HC	5.69E+06	1.14E+09	5.37E+06	1.07E+09
71	HC	4.64E+07	9.28E+09	n.d.	n.d.
72	HC	1.12E+07	2.24E+09	9.25E+04	1.53E+07
73	HC	2.02E+07	4.04E+09	9.68E+06	1.60E+09
74	HC	1.45E+07	2.90E+09	n.d.	n.d.
75	HC	4.18E+07	8.36E+09	n.d.	n.d.
76	HC	6.71E+06	1.34E+09	4.94E+04	7.90E+06
77	HC	2.08E+05	4.16E+07	5.68E+05	9.37E+07
78	HC	1.84E+07	3.68E+09	3.61E+05	5.96E+07
79	HC	2.89E+07	5.78E+09	n.d.	n.d.
80	HC	4.09E+07	8.18E+09	9.72E+06	1.60E+09

* colony forming units or *nusG*-gene equivalents respectively per µL DNA extract and in 0.1 g of stool or 0.1 mL saliva-concentrate;

n.d.: not detected; no total bacterial load could be detected, as those salivary samples were already exhausted during prior experiments.

Table S2: Absolute cell counts and relative abundance (%) of *nusG* targets per sample (1 µL DNA extract of stool) measured by qPCR using different *Fusobacterium nusG*-directed L1 primer pairs.

	NusG1-F/NusG1-R			NusG2a-F/NusG5-R			NusG2a-F/NusG6-R			NusG5-F/NusG6-R		
Sample	Cell amount	%	Ct Mean	Cell amount	%	Ct Mean	Cell amount	%	Ct Mean	Cell amount	%	Ct Mean
41	1.87E+04	0.0765	29.0	*	*	*	*	*	*	*	*	*
42	3.07E+03	0.0208	31.5	*	*	*	*	*	*	*	*	*
43	3.37E+02	0.0110	34.6	*	*	*	*	*	*	*	*	*
44	9.51E+02	0.0073	33.2	*	*	*	*	*	*	*	*	*
45	4.93E+04	0.1947	27.6	4.10E+04	0.1621	30.5	5.70E+04	0.2253	31.4	4.90E+04	0.1938	29.6
46	1.53E+05	1.2370	26.0	1.55E+05	1.2480	28.3	3.55E+05	2.8666	28.0	1.47E+05	1.1856	27.7
47	1.13E+05	0.1332	26.4	7.59E+04	0.0898	29.5	1.85E+05	0.2187	29.2	5.32E+04	0.0629	29.4
48	5.30E+02	0.4530	34.0	*	*	*	*	*	*	*	*	*
49	1.20E+04	0.0181	29.6	1.50E+04	0.0227	32.3	1.36E+04	0.0206	34.1	1.29E+04	0.0194	31.9
50	6.88E+02	0.0056	33.6	*	*	*	*	*	*	*	*	*
51	*	*	*	3.16E+04	5.3351	31.0	*	*	*	*	*	*
52	1.05E+03	1.1923	33.0	*	*	*	*	*	*	*	*	*
53	7.46E+02	0.0018	33.5	*	*	*	*	*	*	*	*	*
54	8.90E+06	28.6224	20.3	5.37E+06	17.2612	22.2	1.74E+07	56.0667	20.7	5.51E+06	17.723	21.4
55	5.96E+05	1.4681	24.1	5.20E+05	1.2801	26.2	1.06E+06	2.6225	25.9	6.38E+05	1.5721	25.1
56	5.84E+02	0.0009	33.8	*	*	*	*	*	*	*	*	*
57	1.57E+05	0.4939	26.0	1.15E+05	0.3614	28.8	2.49E+05	0.7833	28.7	1.47E+05	0.4620	27.7
58	3.81E+03	2.5375	31.2	9.21E+03	6.1393	33.1	3.11E+04	20.7518	32.6	1.30E+04	8.6609	31.8
59	1.43E+05	0.1042	26.1	1.20E+05	0.0873	28.7	1.65E+05	0.1204	29.5	5.46E+04	0.0398	29.4
60	2.91E+03	0.0441	31.6	*	*	*	*	*	*	*	*	*
61	1.06E+03	0.0055	33.0	*	*	*	*	*	*	*	*	*
62	6.45E+05	0.8024	24.0	2.44E+05	0.3036	27.5	9.24E+05	1.1489	26.2	3.84E+05	0.4779	26.0
63	9.39E+02	0.0227	33.2	*	*	*	*	*	*	*	*	*
64	1.13E+03	0.0067	32.9	*	*	*	*	*	*	*	*	*
65	9.11E+04	1.2640	26.7	5.60E+04	0.7769	30.0	7.99E+04	1.1081	30.8	5.79E+04	0.8036	29.3
66	1.24E+03	0.0167	32.8	*	*	*	*	*	*	*	*	*
67	2.21E+03	0.0139	32.0	*	*	*	*	*	*	*	*	*
68	1.35E+03	0.0052	32.7	*	*	*	*	*	*	4.96E+03	0.0189	33.5
69	9.49E+02	0.0041	33.2	6.64E+03	0.0289	33.6				1.45E+04	0.0630	31.6
70	2.04E+03	0.0359	32.1	1.74E+04	0.3057	32.0	2.15E+04	0.3772	33.3	3.06E+04	0.5376	30.4
71	8.82E+02	0.0019	33.3	3.34E+03	0.0072	34.8	*	*	*	9.05E+03	0.0195	32.5
72	1.39E+03	0.0124	32.6	*	*	*	*	*	*	7.11E+03	0.0635	32.9
73	2.06E+04	0.1018	28.8	6.21E+04	0.3073	29.8	6.11E+04	0.3025	31.3	7.05E+04	0.3492	28.9
74	8.53E+02	0.0059	33.3	*	*	*	*	*	*	*	*	*
75	1.29E+03	0.0031	32.7	3.93E+03	0.0094	34.5	*	*	*	6.76E+03	0.0162	33.0
76	1.21E+03	0.0180	32.8	*	*	*	*	*	*	3.66E+03	0.0546	34.0
77	9.89E+02	0.4756	33.1	*	*	*	*	*	*	*	*	*
78	1.13E+03	0.0061	32.9	*	*	*	*	*	*	*	*	*
79	3.52E+02	0.0012	34.6	*	*	*	*	*	*	2.59E+03	0.0090	34.6
80	1.18E+03	0.0029	32.9	*	*	*	*	*	*	6.06E+03	0.0148	33.1

*no detectable amplification after 35 PCR cycles. Samples 41-65: CRC; 66-80: HC

https://doi.org/10.3390/ijms27114865

International Journal of *Molecular Sciences*

MDPI

Table S3: Absolute cell counts per saliva sample (1 µL DNA extract) and relative abundance (%) of *nusG* measured by qPCR using different *Fusobacterium* species-specific *nusG*-directed primer pairs.

	NusG-Fna1			NusG-Fna2			NusG-Fnn			NusG-Fnp			NusG-Fnv		
**Sample**	**Cell amount**	**%**	**Ct Mean**	**Cell amount**	**%**	**Ct Mean**	**Cell amount**	**%**	**Ct Mean**	**Cell amount**	**%**	**Ct Mean**	**Cell amount**	**%**	**Ct Mean**
41	*	*	*	*	*	*	*	*	*	*	*	*	*	*	*
42	*	*	*	1.02E+04	0.0488	28.9	*	*	*	4.89E+04	0.2327	27.3	2.15E+04	0.1023	30.3
43	*	*	*	*	*	*	*	*	*	*	*	*	*	*	*
44	*	*	*	*	*	*	*	*	*	*	*	*	*	*	*
45	3.70E+02	0.0049	30.1	1.55E+04	0.2035	28.3	*	*	*	3.07E+05	4.0237	24.4	*	*	*
46	3.11E+02	0.0005	30.4	1.62E+03	0.0026	31.7	2.75E+03	0.0044	32.463	4.70E+04	0.0757	27.4	*	*	*
47	*	*	*	*	*	*	*	*	*	*	*	*	*	*	*
48	*	*	*	*	*	*	*	*	*	*	*	*	*	*	*
49	3.94E+02	0.0088	30.0	6.34E+03	0.1412	29.6	*	*	*	3.91E+03	0.0870	31.4	4.44E+03	0.0989	33.3
50	*	*	*	*	*	*	*	*	*	*	*	*	*	*	*
51	*	*	*	*	*	*	*	*	*	5.16E+02	0.0010	34.7	*	*	*
52	9.80E+02	0.0043	28.5	3.59E+03	0.0159	30.5	*	*	*	2.64E+03	0.0117	32.1	3.82E+04	0.1688	29.3
53	1.68E+02	0.0011	31.4	1.50E+04	0.0968	30.4	*	*	*	1.50E+04	0.0969	29.3	*	*	*
54	*	*	*	*	*	*	*	*	*	1.09E+03	0.0131	33.5	*	*	*
55	*	*	*	1.79E+03	0.0295	31.5	*	*	*	2.57E+03	0.0423	32.1	*	*	*
56	*	*	*	*	*	*	*	*	*	*	*	*	*	*	*
57	4.49E+03	0.0098	26.0	5.22E+04	0.1138	26.4	*	*	*	2.31E+05	0.5035	24.8	4.13E+04	0.0900	29.1
58	*	*	*	9.50E+02	0.0617	34.6	*	*	*	6.73E+02	0.0437	34.2	*	*	*
59	*	*	*	2.92E+02	0.0012	34.3	*	*	*	3.46E+04	0.1455	27.9	*	*	*
60	*	*	*	*	*	*	*	*	*	*	*	*	*	*	*
61	*	*	*	*	*	*	*	*	*	2.02E+04	0.1038	28.8	*	*	*
62	*	*	*	*	*	*	*	*	*	*	*	*	*	*	*

https://doi.org/10.3390/ijms27114865

63	*	*	*	*	*	*	*	*	*	7.66E+02	0.0056	34.0	*	*	*
64	*	*	*	*	*	*	*	*	*	*	*	*	*	*	*
65	*	*	*	2.53E+03	0.3652	31.0	*	*	*	8.38E+03	1.2073	30.2	*	*	*
70	*	*	*	*	*	*	*	*	*	6.46E+02	0.0120	34.3	*	*	*
72	*	*	*	*	*	*	*	*	*	*	*	*	*	*	*
73	1.47E+03	0.0152	27.9	3.81E+03	0.0393	30.5	*	*	*	7.22E+04	0.7460	26.7	3.82E+04	0.3950	29.3
76	*	*	*	*	*	*	*	*	*	*	*	*	*	*	*
77	6.96E+01	0.0122	32.9	4.22E+02	0.0742	33.8	*	*	*	1.03E+03	0.1818	33.5	*	*	*
78	*	*	*	*	*	*	*	*	*	*	*	*	*	*	*
80	1.55E+02	0.0016	30.4	5.06E+03	0.0521	30.0	*	*	*	2.52E+03	0.0260	32.1	7.74E+03	0.0796	32.2

* no detectable amplification after 35 PCR cycles. Samples 41-65: CRC; 70-80: HC

Table S4: Absolute cell counts and relative abundance (%) of *nusG* targets in L1-positive stool samples (compare with Table S2) measured by qPCR using different *Fusobacterium* species-specific *nusG*-directed primer pairs.

	**NusG-Fna1**			**NusG-Fna2**			**NusG-Fnp**			**NusG-Fnv**		
**Sample**	**Cell amount**	**%**	**Ct Mean**	**Cell amount**	**%**	**Ct Mean**	**Cell amount**	**%**	**Ct Mean**	**Cell amount**	**%**	**Ct Mean**
45	*	*	*	1.57E+04	0.0622	33.6	*	*	*	*	*	*
46	*	*	*	5.19E+04	0.4185	31.2	2.90E+03	0.0234	34.5	2.46E+04	0.1983	31.5
47	3.61E+02	0.0004	27.9	*	*	*	*	*	*	1.36E+04	0.0161	32.5
49	2.82E+01	0.00004	31.1	*	*	*	4.36E+03	0.0066	33.9	*	*	*
54	3.79E+01	0.0001	32.0	3.27E+06	10.5026	23.0	3.99E+05	1.2819	26.5	*	*	*
55	*	*	*	5.99E+05	1.4758	26.4	*	*	*	*	*	*
57	4.92E+03	0.0155	24.5	8.47E+04	0.2664	30.2	2.35E+03	0.0074	34.9	1.08E+04	0.0339	33.0
58	2.12E+02	0.1410	29.1	*	*	*	8.98E+03	5.9869	32.8	*	*	*
59	4.00E+01	0.0000	31.2	9.28E+03	0.0068	34.6	9.55E+04	0.0697	28.8	*	*	*
62	8.22E+01	0.0001	29.8	*	*	*	6.27E+03	0.0078	33.3	5.13E+04	0.0638	30.1
65	1.98E+02	0.0285	28.7	*	*	*	3.06E+03	0.4413	34.4	*	*	*
70	9.32E+01	0.0016	29.6	*	*	*	*	*	*	*	*	*
73	*	*	*	*	*	*	8.25E+03	0.0408	33.2	*	*	*

* no detectable amplification after 35 PCR cycles. Samples 45-65: CRC; 70 and 73: HC; not included is the single result created by NusG-Fnn in sample 62 (CRC): 3.6E+03, 0.0045%.

https://doi.org/10.3390/ijms27114865

International Journal of
Molecular Sciences

MDPI

Table S5: *Fusobacterium* species identified by Sanger sequencing of *nusG* qPCR amplicons in CRC and HC stool samples using *Fusobacterium* L1 primer pairs and a *F. vincentii* species-specific primer pair.

Sample	Group	NusG1-F/NusG1-R	NusG2a-F/NusG6-R	NusG2a-F/NusG-Fnv
45	CRC		*F. pseudoperiodonticum* strain KCOM 1277	
46	CRC		*F. vincentii* strain SB015	*F. vincentii* strain KCOM 3370
47	CRC		*F. vincentii* strain SB015	*F. vincentii* strain KCOM 3370
49	CRC	*F. nucleatum* strain KCOM 3457 (cl. 1)	*F. nucleatum* strain KCOM 3457 (cl. 1)	
54	CRC	*F. animalis* cl. 2 strain KCOM 1279	*F. animalis* cl .2 strain KCOM 1280	
55	CRC	*F. animalis* cl. 2 strain SB033	*F. animalis* cl. 2 strain SB033	
57	CRC	*F. vincentii* strain SB015	*F. vincentii* strain SB015	*F. vincentii* strain KCOM 3370
58	CRC	*F. animalis* cl. 2 strain SB033	*F. animalis* cl. 2 strain KCOM 2952	
59	CRC		*F. polymorphum* strain KCOM 2594	
62	CRC		*F. vincentii* strain SB015	*F. vincentii* strain KCOM 3370
65	CRC	*F. nucleatum* strain KCOM 3457 (cl. 1)	*F. nucleatum* strain KCOM 3457 (cl. 1)	
70	HC		*F. polymorphum* strain KCOM 40A2	
73	HC	*F. vincentii* strain SB015	*F. vincentii* strain SB015	

Species assignment was based on highest sequence similarity to reference sequences using BLAST against the NCBI database. Only samples with successful qPCR amplification and Sanger sequencing results are shown, samples without detectable amplification in qPCR or with low-quality sequences were excluded.

https://doi.org/10.3390/ijms27114865

Table S6: *Fusobacterium* species identified by Sanger sequencing of *nusG* qPCR amplicons in CRC and HC saliva samples using species-specific primer pairs.

Sample	Group	NusG-Fna1	NusG-Fna2	NusG-Fnn	NusG-Fnp	NusG-Fnv
42	CRC		*F. animalis* cl. 2 strain SB045		*F. polymorphum* strain ChDC F306	*F. vincentii* strain KCOM 3370
45	CRC	*F. animalis* strain KCOM 3685 (cl. 1)	*F. animalis* cl. 2 strain SB045		*F. polymorphum* strain ChDC F306	
46	CRC	*F. animalis* strain KCOM 3685 (cl. 1)		*F. nucleatum* strain KCOM 3685	*F. polymorphum* strain SB006	
49	CRC		*F. animalis* cl. 2. strain SB001		*F. polymorphum* strain ChDC F306	*F. vincentii* strain KCOM 3370
52	CRC	*F. animalis* strain KCOM 3685 (cl. 1)	*F. animalis* cl. 2 strain KCOM 3367		*F. vincentii* strain SB037*/*F. polymorphum* strain ChDC F306	*F. vincentii* strain KCOM 3370
53	CRC		*F. animalis* cl.2 strain KCOM 3367		*F. polymorphum* strain CTI-6	
57	CRC				*F. polymorphum* strain CTI-6	
59	CRC				*F. polymorphum* strain CTI-6	
61	CRC				*F. polymorphum* strain CTI-6	
65	CRC		*F. animalis* cl. 2 strain KCOM 3367		*F. polymorphum* strain C48P	
73	HC	*F. animalis* strain KCOM 3685 (cl. 1)	*F. animalis* cl. 2 strain KCOM 3367		*F. polymorphum* strain CTI-6	*F. vincentii* strain KCOM 3370
80	HC		*F. animalis* cl. 2 strain SB020		*F. polymorphum* strain CTI-6	*F. vincentii* strain KCOM 3370

Species assignment was based on highest sequence similarity to reference sequences using BLAST against the NCBI database. Only samples with successful qPCR amplification and Sanger sequencing results are shown, samples without detectable amplification in qPCR or with low-quality sequences were excluded.

*mis-identified/-amplified by the respective PCR-assay.

Table S7: List of reference strains used in this study.

Strain Name	Strain ID
Fusobacterium animalis clade 1	OMI 1486
Fusobacterium animalis clade 2	OMI 1357
Fusobacterium animalis clade 2	OMI 1565
Fusobacterium animalis clade 2	OMI 1566
Fusobacterium animalis clade 2	OMI 1599
Fusobacterium animalis clade 2	OMI 1600
Fusobacterium animalis clade 2	OMI 1601
Fusobacterium canifelinum	OMI 1383
Fusobacterium nucleatum	OMI 72
Fusobacterium nucleatum	OMI 275
Fusobacterium nucleatum	OMI 415
Fusobacterium nucleatum	OMI 1040
Fusobacterium nucleatum	OMI 1309
Fusobacterium nucleatum	OMI 1397
Fusobacterium nucleatum	OMI 1451
Fusobacterium nucleatum	OMI 1605/ATCC 25586
Fusobacterium periodonticum	OMI 269
Fusobacterium polymorphum	OMI 1593
Fusobacterium polymorphum	OMI 1594
Fusobacterium polymorphum	OMI 1595
Fusobacterium polymorphum	OMI 1596
Fusobacterium polymorphum<br>*Fusobacterium varium*	OMI 1597<br>ATCC 8501
Fusobacterium vincentii	OMI 1416
Fusobacterium vincentii	OMI 1598

https://doi.org/10.3390/ijms27114865

Table S8: Demographic data and adenocarcinoma-staging of CRC patients and HC.

Code	Group	Sex	Age*	Smoking	Alcohol consumption	Nutrition diet	Antibiotic usage	Adenocarcinoma-stage
1=41	CRC	female	60	no	no	high meat consumption and rice	no	TNM 0-1 (early)
2=42	CRC	male	60	yes	no	high meat, low fruit, vegtables	no	TNM 0-1 (early)
3=43	CRC	female	51	no	no	all foods	no	TNM 0-1 (early)
4=44	CRC	male	84	no	no	red meat, fruit, but low vegetables	no	TNM 0-1 (early)
5=45	CRC	female	57	no	no	all foods	no	TNM 0-1 (early)
6=46	CRC	male	57	no	no	meat, rice, traditional Iranian food	no	TNM 0-1 (early)
7=47	CRC	male	72	yes	no	low vegetables, with and red meat	no	TNM 0-1 (early)
8=48	CRC	male	68	yes	yes	all foods	no	TNM 0-1 (early)
9=49	CRC	female	62	no	no	all foods	no	TNM 0-1 (early)
10=50	CRC	female	62	no	no	all food, fruits and vegtables, low meat	no	TNM 0-1 (early)
11=51	CRC	male	25	yes	no	all food, fast food two times a week	no	TNM 0-1 (early)
12=52	CRC	female	45	no	no	traditional Iranian food, meat consumption	no	TNM 0-1 (early)
13=53	CRC	male	63	no	no	all food	no	TNM 0-1 (early)
14=54	CRC	female	56	no	no	all food, high fruit	no	TNM 0-1 (early)
15=55	CRC	female	74	no	no	meat (white and red)	no	TNM 0-1 (early)
16=56	CRC	male	62	yes	no	all food	no	TNM 0-1 (early)
17=57	CRC	male	41	yes	no	all food	no	TNM 0-1 (early)
18=58	CRC	female	40	no	no	all food, white meat	no	TNM 0-1 (early)
19=59	CRC	male	83	no	no	all food, not vegetables, fruit	no	TNM 0-1 (early)
20=60	CRC	male	65	no	no	all food	no	TNM 0-1 (early)
21=61	CRC	male	73	no	no	all food,	no	TNM 0-1 (early)
22=62	CRC	female	49	no	no	all food	no	TNM 0-1 (early)
23=63	CRC	male	68	no	no	all food,	no	TNM 0-1 (early)
24=64	CRC	female	70	no	no	all food, meat and chicken	no	TNM 0-1 (early)
25=65	CRC	female	25	no	no	vegetables, fruit, rice, low meat	no	TNM 0-1 (early)
26=66	HC	male	31	no	no	all food	no	n.a.
27=67	HC	male	30	no	no	all food, high protein, high fiber	no	n.a.
28=68	HC	male	60	no	no	all food	no	n.a.
29=69	HC	female	42	no	no	all food, high fruit and vegtables	no	n.a.
30=70	HC	male	55	no	no	all food	no	n.a.
31=71	HC	female	36	no	no	all food	no	n.a.
32=72	HC	female	25	no	no	all food	no	n.a.
33=73	HC	male	36	no	no	all food	no	n.a.
34=74	HC	female	53	no	no	low meat, enough vegtables	no	n.a.
35=75	HC	male	62	no	no	all food	no	n.a.
36=76	HC	female	38	no	no	all food	no	n.a.
37=77	HC	male	37	no	no	see products, fish	no	n.a.
38=78	HC	male	31	no	no	high fruit and vegetables	no	n.a.
39=79	HC	male	31	no	no	all food	no	n.a.
40=80	HC	male	25	no	no	low meat, enough vegetables	no	n.a.

* at time of sampling
n.a. not applicable

Disclaimer/Publisher's Note: The statements, opinions and data contained in all publications are solely those of the individual author(s) and contributor(s) and not of MDPI and/or the editor(s). MDPI and/or the editor(s) disclaim responsibility for any injury to people or property resulting from any ideas, methods, instructions or products referred to in the content.
